# Identification of miRNAs of *Strongyloides stercoralis* L1 and iL3 larvae isolated from human stool

**DOI:** 10.1038/s41598-022-14185-y

**Published:** 2022-06-15

**Authors:** Elena Pomari, Giovanni Malerba, Laura Veschetti, Alessandra Franceschi, Lucas Moron Dalla Tor, Michela Deiana, Monica Degani, Manuela Mistretta, Cristina Patuzzo, Andrea Ragusa, Antonio Mori, Zeno Bisoffi, Dora Buonfrate

**Affiliations:** 1 Department of Infectious-Tropical Diseases and Microbiology, IRCCS Sacro Cuore Don Calabria Hospital, Negrar di Valpolicella, Verona, Italy; 2 Department of Neurosciences, Biomedicine and Movement Sciences, University of Verona, Verona, Italy; 3 Department of Medicine and Surgery, University of Parma, Parma, Italy; 4 Department of Diagnostics and Public Health, University of Verona, Verona, Italy

**Keywords:** Computational biology and bioinformatics, Molecular biology, Zoology

## Abstract

Strongyloidiasis is a neglected tropical disease caused by the soil-transmitted nematode by *Strongyloides stercoralis*, that affects approximately 600 million people worldwide. In immunosuppressed individuals disseminated strongyloidiasis can rapidly lead to fatal outcomes. There is no gold standard for diagnosing strongyloidiasis, and infections are frequently misdiagnosed. A better understanding of the molecular biology of this parasite can be useful for example for the discovery of potential new biomarkers. Interestingly, recent evidence showed the presence of small RNAs in Strongyloididae, but no data was provided for *S. stercoralis*. In this study, we present the first identification of miRNAs of both L1 and iL3 larval stages of *S. stercoralis*. For our purpose, the aims were: (i) to analyse the miRNome of L1 and iL3 *S. stercoralis* and to identify potential miRNAs of this nematode, (ii) to obtain the mRNAs profiles in these two larval stages and (iii) to predict potential miRNA target sites in mRNA sequences. Total RNA was isolated from L1 and iL3 collected from the stool of 5 infected individuals. For the miRNAs analysis, we used miRDeep2 software and a pipeline of bio-informatic tools to construct a catalog of a total of 385 sequences. Among these, 53% were common to *S. ratti*, 19% to *S. papillosus*, 1% to *Caenorhabditis elegans* and 44% were novel. Using a differential analysis between the larval stages, we observed 6 suggestive modulated miRNAs (STR-MIR-34A-3P, STR-MIR-8397-3P, STR-MIR-34B-3P and STR-MIR-34C-3P expressed more in iL3, and STR-MIR-7880H-5P and STR-MIR-7880M-5P expressed more in L1). Along with this analysis, we obtained also the mRNAs profiles in the same samples of larvae. Multiple testing found 81 statistically significant mRNAs of the total 1553 obtained (FDR < 0.05; 32 genes expressed more in L1 than iL3; 49 genes expressed more in L3 than iL1). Finally, we found 33 predicted mRNA targets of the modulated miRNAs, providing relevant data for a further validation to better understand the role of these small molecules in the larval stages and their valuein clinical diagnostics.

## Introduction

*Strongyloides stercoralis* is a soil-transmitted helminth affecting about 600 million people worldwide^1^. This parasite leads to chronic infection in the human host who, in the event of immunosuppression, can develop a life-threatening syndrome (hyperinfection or disseminated disease)^2,3^. There is no gold standard for diagnosing strongyloidiasis, and infections are frequently misdiagnosed. In the last years, the ‘omic’ approaches have been used to provide relevant information to better characterize the molecular mechanisms involved in the biology and parasitism of this nematode. The *S. stercoralis* life cycle is complex, alternating free-living and parasitic cycles, and involving autoinfection^4,5^. Briefly, the free-living infective filariform larvae (iL3) in soil can penetrate the human skin, and typically migrate to the small intestine via the lungs. In the small intestine the iL3 become adult female worms, that produce eggs. The eggs embryonate via parthenogenesis and hatch into rhabditiform larvae (L1). Some L1 are excreted with stool, while others transform into filariform larvae, which can re-infect the individual leading to chronic infection (“auto-infective cycle”). The L1 (mix of males and females) and iL3 (only females) can be distinguished by their morphology and behavior. In particular, L1 have a short buccal canal and their energy consumption is mainly related to feeding and growth, while iL3 have a closed buccal cavity and a thick cuticle that enables them to survive in the external environmental and into the host’s gastrointestinal tract^6^.

In the field of transcriptomics, previous studies that characterized the mRNA profiles of *S. stercoralis*, contributed to understanding the role of transcripts in the evolution of this nematode*.* The majority of these studies performed mRNA profiling of iL3 and adults, using PV001 and UPD strains isolated from non-human stool^7–11^. For example, Stoltzfus et al.^7^ provided PV001 strain transcriptomics data sets of seven developmental stages including L1 and iL3 isolated from gerbil. Ramanathan et al*.*^12^ described DNA microarray for *S. stercoralis* UPD strain isolated from dog stool to compare iL3 with L1, suggesting a high degree of specificity between these stages. RNAseq data sets of *S. stercoralis* iL3 from a stool sample of one infected human individual only were published by Marcilla et al.^13^, and Rodpai et al.^14^ analysed the effect of dexamethasone on transcriptome profiles of pooled adult worms isolated from stool of infected patients. In the present study we investigated miRNAs of two larval stages L1 and iL3 through RNAseq approach. Small RNAs have been studied extensively in nematodes^15,16^. Using RNAseq, miRNAs were investigated in Strongyloididae including *S. ratti* and *S. papillosus*^17,18^, natural parasites of *Rattus norvegicus* and ruminants, respectively. The close relation of these species to *S. stercoralis*, suggested that this type of small RNAs might also be expressed in *S. stercoralis*.

Aims of this work were to investigate for the first time the miRNome of *S. stercoralis*, and to identify potential miRNAs specific of this nematode. Moreover, the analysis of mRNAs from the same samples, allowed us to predict potential targeting between the identified miRNAs and mRNAs.

## Results

### Identification of miRNAs and differential expression of mature miRNAs

**Figure 1:**
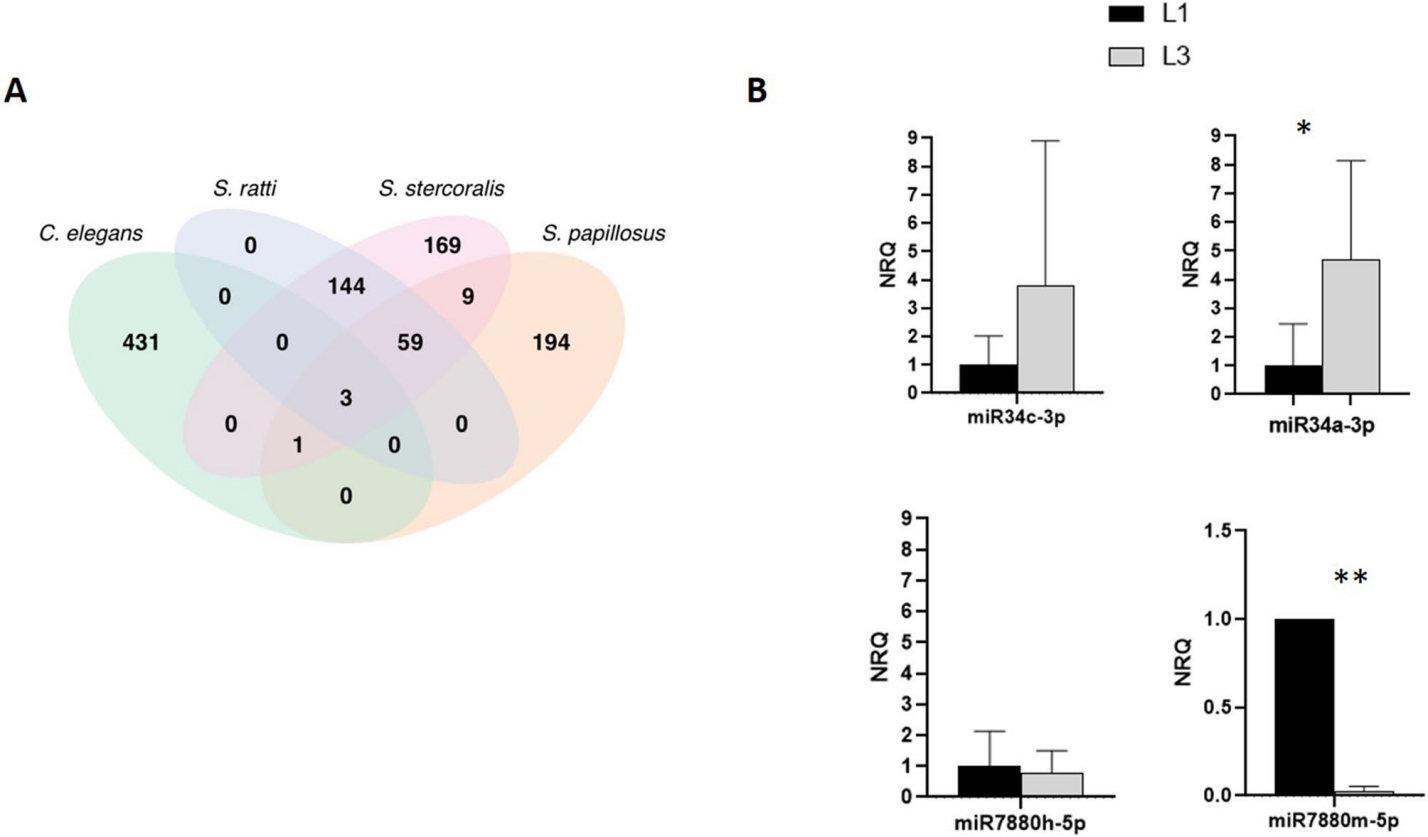
miRNAs identification for *S. stercoralis*. (**A**) Known and novel miRNAs were identified in *S. stercoralis* compared to *S. ratti, S. papillosus* and *C. elegans*. (**B**) RT-qPCR was used to verify the expression and modulation of miRNAs between L1 and iL3. Data are expressed as NRQ. For the comparison analysis, L1 expression value of each miRNA was set equal to 1. P value < 0.05*; < 0.01**.

Predictions of mature and precursor miRNAs were made using all the reads of all the 8 samples (iL3 and L1). We estimated 385 and 208, mature and precursor unique miRNA sequences, respectively (see Supplementary Material 1). Some miRNA mature sequences that were detected in the analyses showed 100% identity with miRNAs of *S. ratti* (n = 206), of *S. papillosus* (n = 72) and of *Caenorhabditis elegans* (n = 4). Novel detected miRNAs did not show any match with either *S. ratti, S. papillosus* or *C. elegans* (n = 169). See Fig. 1A. Differential gene expression analysis was only performed on mature miRNAs (n = 294) that presented 20 or more normalized counts from the sum of the counts from all 8 samples (see Supplementary Material 2). Comparing the miRNA expression in L1 and iL3, we observed 6 modulated miRNAs (|log2FoldChange|> 1 and nominal p-value < 0.05); 4 miRNAs were expressed more in iL3 than L1 (STR-MIR-34A-3P, STR-MIR-8397-3P, STR-MIR-34B-3P, STR-MIR-34C-3P); 2 miRNAs expressed more in L1 than iL3 (STR-MIR-7880H-5P, STR-MIR-7880M-5P). However, miRNA expression between L1 and iL3 was not significantly different when correction for multiple testing was applied.

### RT-qPCR verification of miRNAs

miRNAs with the highest and medium–high differential expression levels between L1 and iL3 (see Supplementary Material 2) were then tested by RT-qPCR, as an independent gene expression measurement method. As reported in Fig. 1B, STR-MIR-34A-3P, STR-MIR-34C-3P were up-regulated in iL3, while STR-MIR-7880H-5P and STR-MIR-7880M-5P were expressed more in L1 according to RNAseq. Differences in RT-qPCR gene expression values between L1 and iL3 stages were tested using a one-tail Student's t-test. Results were validated for STR-MIR-34A-3P (p-value = 0.041) and STR-MIR-7880M-5P (p-value = 0.0005) whereas no significant differences were observed for STR-MIR-34C-3P and STR-MIR-7880H-5P. No amplification signal was detected in the negative controls (data not shown).

### Functional annotation of *S. stercoralis* genes

The available *S. stercoralis* annotations, which comprise 9290 annotated sequences, still include a significant number of hypothetical proteins or proteins of unknown function (n = 5754). To try and assign a function to these, we functionally annotated the aminoacidic sequences of *S. stercoralis* proteins (ver. WBPS14) by transferring functional information using precomputed orthologous groups and phylogenies from the eggNOG database. The sequences were classified into 24 eggNOG functional sub-categories; functional description and number of genes belonging to each sub-category are reported in Table 1. Starting from 13,098 proteins, a total of 9986 sequences resulted as functionally annotated when combining both already available WBPS14 annotations and eggNOG annotations. Nonetheless 5058 (38.6%) sequences remain of unknown function, including: 3112 (23.8%) with no annotation and 1946 (14.8%) annotated as “function unknown”.

**Table 1:** Functional annotation of *Strongyloides stercoralis* sequences. Functional description and number of genes belonging to each eggNOG sub-category are reported.

Functional category description	Number of genes	%
Amino acid transport and metabolism	236	2.36
Carbohydrate transport and metabolism	412	4.13
Cell cycle control, cell division, chromosome partitioning	232	2.32
Cell motility	8	0.08
Cell wall/membrane/envelope biogenesis	73	0.73
Chromatin structure and dynamics	164	1.64
Coenzyme transport and metabolism	66	0.66
Cytoskeleton	319	3.19
Defense mechanisms	103	1.03
Energy production and conversion	343	3.43
Extracellular structures	193	1.93
Function unknown	1946	19.49
Inorganic ion transport and metabolism	422	4.23
Intracellular trafficking, secretion, and vesicular transport	577	5.78
Lipid transport and metabolism	354	3.54
Nuclear structure	33	0.33
Nucleotide transport and metabolism	120	1.20
Post-translational modification, protein turnover, chaperones	1038	10.39
Replication, recombination and repair	270	2.70
RNA processing and modification	338	3.38
Secondary metabolites biosynthesis	159	1.59
Signal transduction mechanisms	1488	14.90
Transcription	726	7.27
Translation, ribosomal structure and biogenesis	366	3.67
Total	9986	100

### Differential gene expression

**Figure 2:**
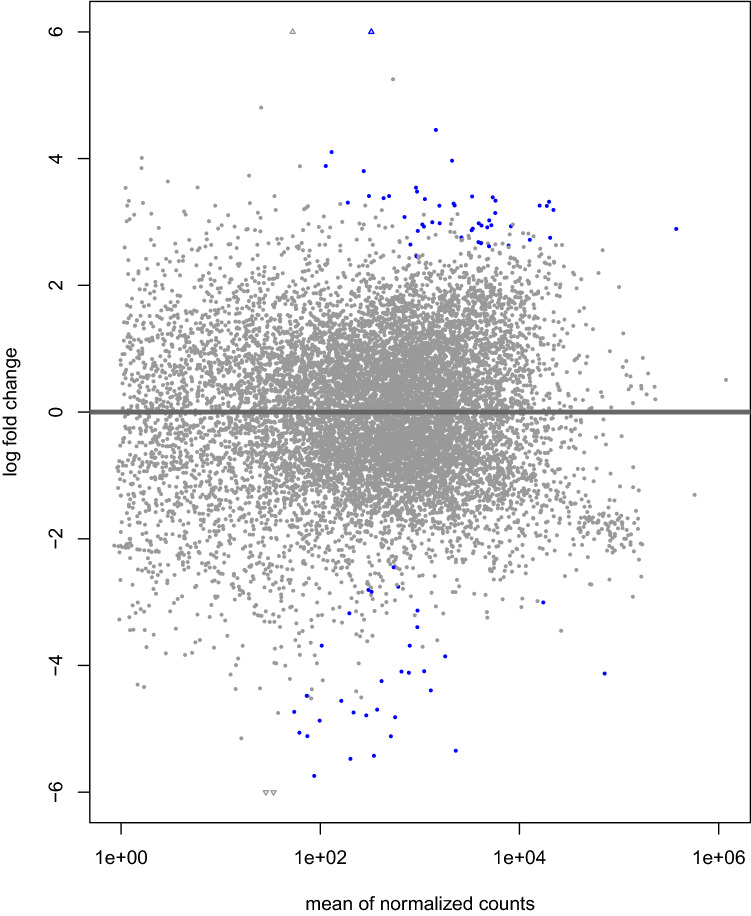
Scatter plot of mRNA log2 fold change of iL3-vs-L1 expression values (on the y-axis) versus the mean of normalized counts (on the x-axis). Dots represent individual transcripts. Blue dots indicate the 81 modulated genes (p-adjusted ≤ 0.05).

Comparing the transcripts expression in L1 and iL3, we found 1553 modulated genes (|log2FoldChange|> 1 and nominal-pvalue < 0.05; 802 genes were more expressed in L1 than iL3; 751 genes were more expressed in L3 than iL1) as shown in Fig. 2 (see Supplementary_Material_3 for details and Supplementary Fig. S1). When accounting for multiple testing, the differential expression of only 81 of the 1553 genes was statistically significant (FDR < 0.05; 49 genes more expressed in L1 than iL3; 32 genes more expressed in iL3 than L1). Because L1 and iL3 larvae represent different larval stages of the same worm, statistical tests were performed on paired data obtained from the same individual.

### Gene set enrichment analysis

To determine whether *S. stercoralis* gene sets showed biased expression in L1 and iL3, a total number of 32 gene sets were created: each of the 24 eggNOG sub-category obtained by functional annotation was considered as a gene set; 4 gene-sets were manually generated to include genes known to be differentially expressed from the literature^12^ (i.e., a heat-shock protein encoding gene set, a putative antigen gene set, a prokaryotes heat-shock protein gene set, and a gene set containing genes encoding products known to be immunoreactive in *S. stercoralis*-infected humans); and finally, 4 randomly generated gene sets to test the method robustness were included in the analysis. Thirty of the 32 gene sets met the criteria for inclusion into the Gene Set Enrichment Analysis (GSEA), while “Function unknown” and “Cell motility” gene sets were excluded. Of these 30 gene sets, 6 were significantly (p < 0.05) enriched in the iL3 phenotype, namely: cytoskeleton, extracellular structures, immunoreactive genes, inorganic ion transport and metabolism, signal transduction mechanisms, and transcription. Other 13 gene sets were significantly (p < 0.05) enriched in the L1 phenotype: amino acid transport and metabolism; carbohydrate transport and metabolism; cell cycle control, cell division, chromosome partitioning; coenzyme transport and metabolism; energy production and conversion; lipid transport and metabolism; nuclear structure; nucleotide transport and metabolism; post-translational modification, protein turnover, chaperones; replication, recombination and repair; RNA processing and modification; secondary metabolites biosynthesis, transport and catabolism; translation, ribosomal structure and biogenesis. Further details regarding significantly enriched gene sets are reported in Table 2 (see also Supplementary Material 4 and Supplementary Fig. S2A,B).

**Table 2:** Gene-set enrichment analysis in L1 and iL3.

Larval stage	Gene-set	Size	ES	NES	NOM p-val	FDR q-val
L1	Amino acid transport and metabolism	236	− 0.24906003	− 16.352.892	0.0	0.0090
L1	Carbohydrate transport and metabolism	412	− 0.1939296	− 13.788.581	0.0	0.045
L1	Cell cycle control, cell division, chromosome partitioning	232	− 0.2724666	− 17.923.241	0.0020120724	0.0034
L1	Coenzyme transport and metabolism	66	− 0.39813814	− 20.189.772	0.0	2.24E + 03
L1	Energy production and conversion	343	− 0.3508425	− 24.511.664	0.0	0.0
L1	Lipid transport and metabolism	354	− 0.2494798	− 17.342.019	0.0	0.0041
L1	Nuclear structure	33	− 0.552677	− 24.175.467	0.0	0.0
L1	Nucleotide transport and metabolism	120	− 0.27925467	− 16.489.633	0.002016129	0.0096
L1	Post-translational modification, protein turnover, chaperones	1038	− 0.18970026	− 14.864.627	0.0	0.024
L1	Replication, recombination and repair	270	− 0.2641072	− 17.816.738	0.0	0.0031
L1	RNA processing and modification	338	− 0.310632	− 21.683.147	0.0	0.0
L1	Secondary metabolites biosynthesis, transport and catabolism	159	− 0.25706917	− 15.833.042	0.0020833334	0.017
L1	Translation, ribosomal structure and biogenesis	366	− 0.5848706	− 40.967.445	0.0	0.0
iL3	Cytoskeleton	319	0.3042337	21.056.857	0.0	4.74E + 03
iL3	Extracellular structures	193	0.27855292	17.662.898	0.0	0.003
iL3	Immunoreactive genes encoding antigens recognized by sera from patients infected with S stercoralis	22	0.5554037	21.882.079	0.0020920502	3.44E + 00
iL3	Inorganic ion transport and metabolism	422	0.2727433	19.638.805	0.0	5.60E + 03
iL3	Signal transduction mechanisms	1488	0.33428338	26.924.367	0.0	0.0
iL3	Transcription	726	0.26496243	20.102.034	0.0	5.17E + 03

*ES* enrichment score, *NES* normalised enrichment score (accounts for differences in gene set size), *NOM p-val* nominal p-value, *FDR q-val* false discovery rate q-value.

### mRNA 3′-UTR and miRNA target prediction

Messenger RNA sequences were analysed to predict three prime untranslated regions (3′-UTR), which are potential targets of miRNA. A total of 6480 3′-UTR were predicted and considered as candidate targets for the 6 modulated miRNAs (see previous section). Following miRNAs target analysis, a total of 33 possible targets were identified (Supplementary Material 5) including, transcripts involved in transcription, translation, metabolism and energy production, cytoskeleton rearrangements, signal transduction, and 8 transcripts of unknown function. Six transcripts of the 33 putative targets showed to be differentially expressed between L1 and iL3 (at nominal p-value < 0.05) (Table 3 and Supplementary Material 5). STR-MIR-34C-3p was reported to putatively target thirteen of the 33 transcripts. Moreover, out of the 33 target transcripts, six had a significant level of differential expression between L1 and iL3 (p-value < 0.05). Among these 6 transcripts, the microtubule-binding protein MIP-T3 and another transcript without annotation (SSTP_0000331600) resulted more expressed in iL3 with FC > 1 and putatively targeted by STR-MIR-34C-3p.

**Table 3:** List of top significant predicted targeting between mRNAs and miRNAs of *S. stercoralis*. Rank of match indicates the position of the match in the ranked list of matches (ordered by strength of match) outputted by the software PITA; multiple ranks indicate multiple matching between miRNA and mRNA. ^a^Differentially expressed putative targets at nominal p-value < 0.05.

mRNA eggNOG annotations	Significative miRNA match	Rank of match
Microtubule-binding protein MIP-T3^a^	str-miR-34C-3p	11
Kelch-like^a^	str-miR-34b-3p	1
Pyruvate kinase^a^	str-miR-7880m-5p	1
Ribosomal protein L7/L12 C-terminal domain^a^	str-miR-34A-3p str-miR-34C-3p	11 2
B-cell receptor-associated protein 31-like^a^	str-miR-7880h-5p str-miR-34A-3p	11 5
NA (SSTP_0000331600)^a^	str-miR-34C-3p	4
Fibronectin type III domain	str-miR-7880h-5p	5, 6, 7, 8, 9
Neural proliferation differentiation control-1 protein (NPDC1)	str-miR-7880m-5p	2
ENTH domain	str-miR-34C-3p	3
Catalytic subunit of the tRNA-splicing ligase complex that acts by directly joining spliced tRNA halves to mature-sized tRNAs by incorporating the precursor-derived splice junction phosphate into the mature tRNA as a canonical 3′,5′-phosphodiester. May act as a RNA ligase with broad substrate specificity, and may function toward other RNAs (By similarity)	str-miR-34b-3p	7
DZF domain	str-miR-34A-3p str-miR-34C-3p	6 13
Solute carrier family 25 (pyrimidine nucleotide carrier	str-miR-34b-3p	6
HCO3-transporter family	str-miR-8397-3p	1
Domain of unknown function (DUF1768)	str-miR-7880h-5p	10
Specifically acetylates 'Lys-40' in alpha-tubulin on the lumenal side of microtubules. May affect microtubule stability and regulate microtubule dynamics. Required for the maintenance of touch receptor neurons. Mutants greatly reduce the touch responsiveness of the worm (By similarity)	str-miR-34C-3p	10
Glucosidase II beta subunit-like protein	str-miR-7880h-5p	12
WD40	str-miR-34A-3p str-miR-34C-3p	1 1
Ubiquitin carboxyl-terminal hydrolase	str-miR-7880h-5p	4
UTP-glucose-1-phosphate uridylyltransferase	str-miR-7880h-5p	3
Transmembrane amino acid transporter protein	str-miR-7880h-5p	13
C2 domain	str-miR-7880h-5p	1
Acyltransferase	str-miR-34C-3p	14
Brix	str-miR-34b-3p str-miR-34C-3p	4 5, 12
Glutathione S-transferase, N-terminal domain	str-miR-34A-3p	2, 3, 4
Dynamin central region	str-miR-34C-3p	7
ZIP Zinc transporter	str-miR-7880h-5p	2
WD domain, G-beta repeat	str-miR-34C-3p	9
C4	str-miR-34A-3p	10
MreB/Mbl protein	str-miR-34C-3p	8
UBX domain protein 7	str-miR-34b-3p	2
RNA binding activity-knot of a chromodomain	str-miR-34b-3p	3, 5
NA (SSTP_0000519100.1)	str-miR-34C-3p	6
Protein kinase, cAMP-dependent, catalytic	str-miR-34A-3p	7, 8, 9

## Discussion

In this study, we obtained for the first time the miRNA profile of *S. stercoralis* L1 and iL3 isolated from human stool. The analysis of transcriptional regulation mechanisms and the differences in miRNome and miRNAs expression profiles between the infective and non-infective larvae in particular, may represent a starting point for an increased understanding of the biological operating mechanism of these molecules. In animals, miRNAs demonstrated to play a role in development and differentiation^19,20^. Small RNAs have also been investigated in nematodes; for instance, miRNAs were demonstrated to be responsible for the developmental switching of *C. elegans*^21–25^. Moreover, miRNAs have been investigated in Strongyloididae including *S. ratti* and *S. papillosus*^17,18^, but data for *S. stercoralis* were not available yet.

First, in our study we used a pipeline of bio-informatics tools in order to classify sequences as miRNAs or not, on the basis of some characteristics considered typical of miRNAs: the mapping of the precursor on specific genomic locations as well as the prediction of the potential miRNA precursor^26^. We collected a catalog of 385 miRNA sequences, of which 44% (169/385) were novel, while others were shared with either the catalog of the miRNAs of *S. ratti* (53%, 206/385), *S. papillosus* (19%, 72/385) or *C. elegans* (1%, 4/385).

After the identification of miRNAs, we investigated their differential expression between L1 and iL3. We identified a total of 6 suggestive modulated miRNAs (nominal p-value < 0.05) common to *S. ratti* reported in a previous work^17^, but no data are available on the function of these miRNAs. Further investigation is needed to better understand their biological roles and possible association with the developmental switching between larval states.

miRNAs demonstrated regulatory roles in modulating the expression of specific mRNAs in many organisms^27–30^. In line with our aims, using RNAseq we investigated the mRNAs targets of the miRNAs that we identified. We obtained mRNAs profiles that were previously reported to characterize the transcriptome of different stages of *S. stercoralis*. From the GSEA, L1 appeared to be transcriptionally more active than iL3^12^. In particular, we found up-regulation of metalloproteases in iL3 such as astacin that can be involved in skin penetration^31–33^ and in the production of antigens, which have been found in sera of people with strongyloidiasis^34^. Moreover, proteins of the astacin family have been identified and extensively investigated in Strongyloididae, and are important to distinguish parasitic from non-parasitic lineages^33,35–37^. Among the mRNAs, we investigated the potential 3′-UTR targets of the 6 suggestive modulated miRNAs. We found 33 predicted target transcripts involved in transcription, translation, metabolism and energy production, cytoskeleton rearrangements and signal transduction. These 33 transcripts significantly modulated between L1 and iL3. In particular, STR-MIR-34C-3p matched the majority of the transcripts (astacin or metalloproteases were not found). We speculate that a possible regulatory relationship between the differentially expressed miRNAs and their differentially expressed putative targets exists, based on available evidence in literature of orthologs of other species of Strongyloides, and also of *C. elegans*^38^. For instance, MIP-T3 was over-expressed in iL3 and putatively targeted by STR-MIR-34C-3p. MIP-T3 is a conserved protein across species from worms to humans, and plays a critical role in *C. elegans* in assembling functional intraflagellar transport complexes in biogenesis of sensory cilia^39^. Comparisons of the *C. elegans* amphid neuroanatomy to those of free-living and parasitic nematodes such as *S. stercoralis*, have shown that amphid neurons are broadly conserved^40^. *S. stercoralis* has evolved a lamellar morphology for its putative thermosensory neuron ALD hypothesizing a mediation of thermotaxis by the skin-penetrating infective larva^41^ and with possible functional similarity to AFD neurons in *C. elegans*^42^. These data suggest a role of STR-MIR-34C-3p in the nervous system patterns of *S. stercoralis*. Moreover, we found pyruvate kinase (pyk) putatively targeted by STR-MIR-7880M-5p. Pyk is an important enzyme involved in the subpathway of glycolysis and it was reported with low expression in parasitic females of *S. ratti* suggesting their anaerobic energy metabolism^43^. In our samples of *S. stercoralis,* pyk was expressed less in iL3 than in IL1, thus indicating a possible role of STR-MIR-7880M-5p in enzymatic and metabolic transition of different stages. Overall, our findings of miRNA and putative targets warrant further investigations to better understand the role of miRNAs in *S. stercoralis*. First, it will be necessary to verify the predicted mRNA targets and the potential effect of miRNAs on their modulation. Moreover, studying the potential interaction between *S. stercoralis* miRNAs and human target transcripts could provide new insights in the host–pathogen interaction for strongyloidiasis.

### Limitations of the study

In presenting these results, we are aware that a larger sample size would be needed to achieve statistically reliable results. However, based on a robust approach, we believe that our findings are relevant for future analyses.

## Conclusions

We presented the transcriptome and the first miRNome profiles of both L1 and iL3 larval stages of *S. stercoralis* isolated from human stool. Overall, our findings provide insights for future ‘omics’ investigations of *S. stercoralis* and the development of novel approaches for detection of this parasite. Further studies are required to verify miRNAs that we identified in a larger sample size and to confirm whether miRNAs are good targets for the diagnosis of strongyloidiasis. Moreover, the combination of transcriptomic and proteomic data could allow for better characterization of differential gene expression and critical pathways in the different stages of *S. stercoralis*.

## Methods

### Sample collection and isolation of larvae

**Figure 3:**
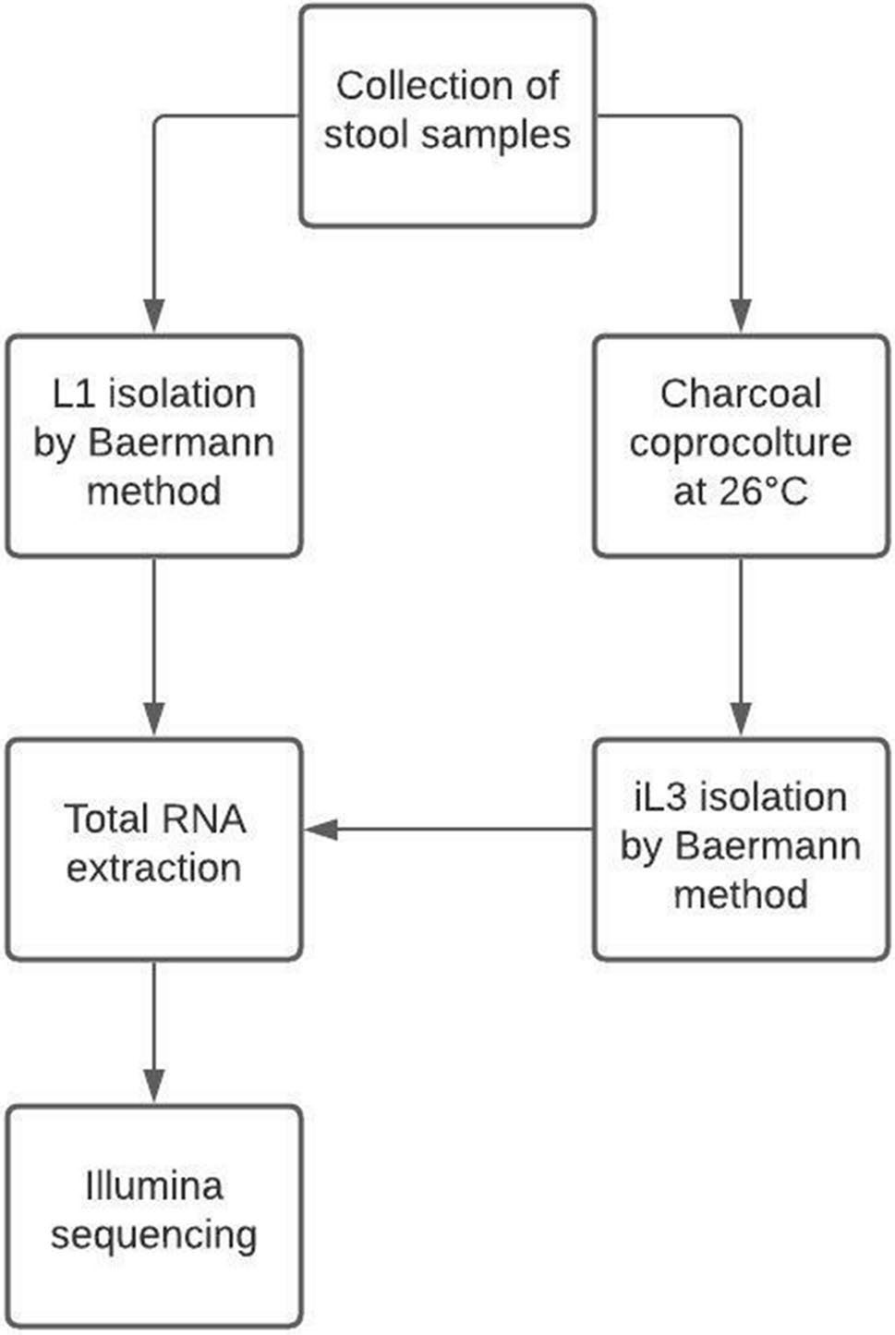
Flowchart of the study.

**Figure 4:**
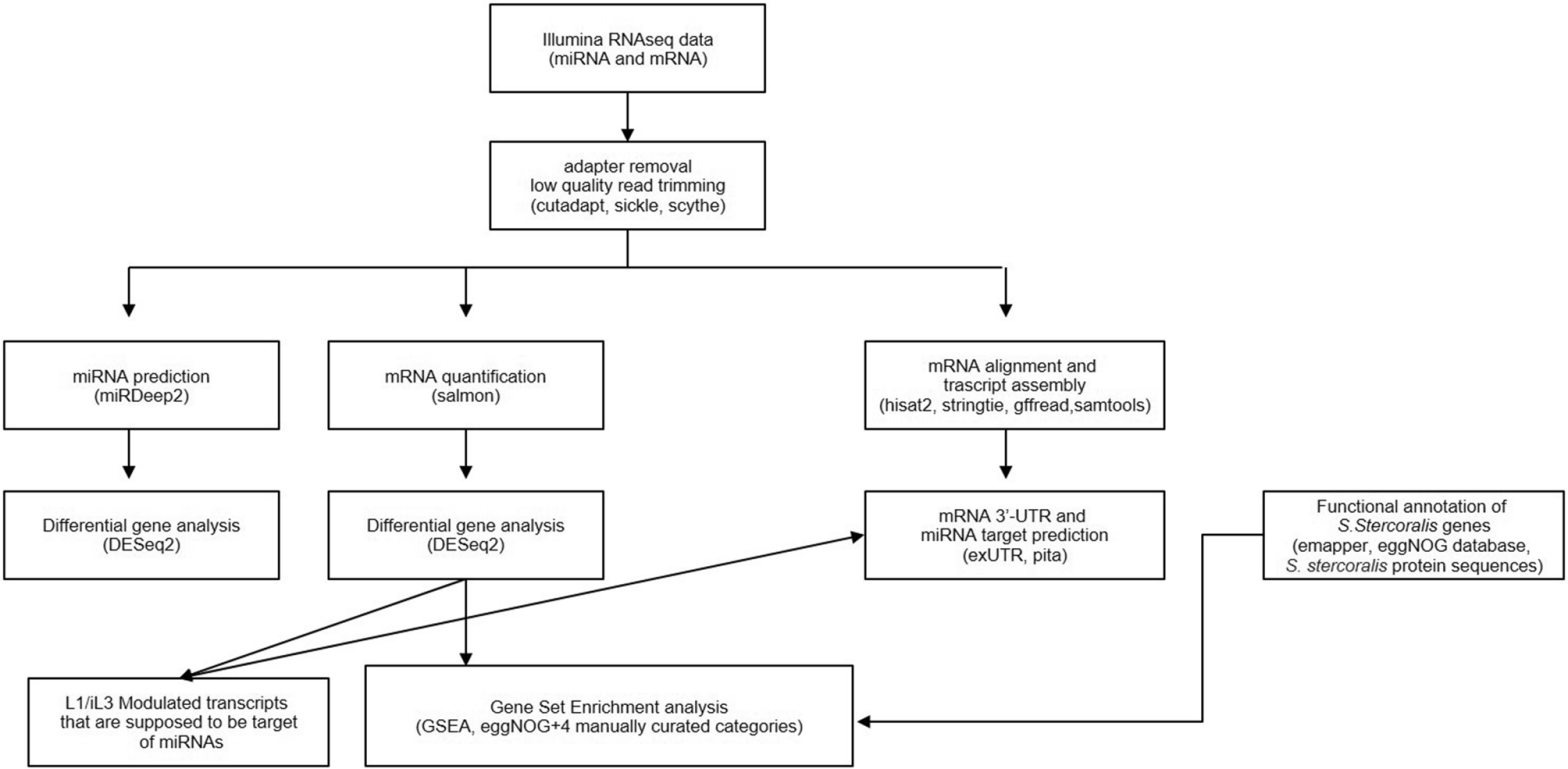
Flowchart of mRNASeq and miRNASeq bioinformatic analysis.

Faecal samples (n total = 8) were obtained at the IRCCS Sacro Cuore Don Calabria Hospital, Negrar di Valpolicella, Verona (Italy) from a total of five patients from sub-Saharan Africa, infected with *S. stercoralis.* Precisely, we obtained paired L1 and iL3 only from 3 patients because of high parasitaemia*.* The isolation of larvae was performed by Baermann funnel technique. L1 larvae (n total = 3) were recovered from freshly deposited stool samples; iL3 larvae (n total = 5) were recovered after seven days from the charcoal coproculture at 26 °C. All L1 and iL3 larvae were harvested and concentrated by centrifugation for 10 min at 1000 g, washed three times in 1 mL of phosphate buffered saline (PBS) pH 7.2 containing antibiotic/antimycotic cocktail (100 U/mL penicillin, 100 μg/mL streptomycin and 0.25 μg/mL amphotericin B). Decontaminated larvae were subsequently stored in Qiazol reagent (Qiagen) at − 80 °C. The flowchart of the present study is reported in Figs. 3 and 4.

### Isolation of total RNA from larvae

Total RNA was extracted by thawing at room temperature the samples of L1 (2000 larvae) and iL3 (2000 larvae). Then, a mechanical disruption was performed by adding ceramic beads and using the MagNA Lyser Instrument (Roche). Samples were purified using RNeasy mini kit (Qiagen) following the manufacturer's protocol. The RNA was quantified with Nanodrop Spectrophotometer (Thermo Scientific) and the RNA integrity (RIN > 8) was evaluated using capillary electrophoresis with 2100 Bioanalyzer (Agilent).

### *S. stercoralis* polyadenylated mRNA and miRNA library preparation and sequencing

RNA-seq libraries were generated from 1000 ng of total RNA using the TruSeq Stranded mRNA Library Prep Kit according to the manufacturer’s protocol (Illumina, Inc., http://www.illumina.com). In each sample polyadenylated coding mRNA was isolated using olido-dT beads and reverse transcribed using random primers before generating final stranded libraries.

miRNA libraries were prepared from 300 ng of total RNA using QIAseq miRNA Library Kit (Qiagen). Briefly, 3′ and 5′ adapters were ligated to mature miRNAs and used for cDNA synthesis and PCR amplification. The quality of the libraries was assessed using Fragment Analyzer (Agilent).

The average library size (= length of fragments including adapters) was 320 bp and 180 bp for RNAseq and miRNASeq, respectively. The numbers of cycles of amplification for the libraries were 12 and 16 for RNAseq and miRNASeq, respectively. The adapters used for multiplexing were the ones included in the Illumina TruSeq Index Adapters (Index 1–27) kit and QIAGEN QIAseq miRNA NGS 48 Index kit, for RNAseq and miRNASeq, respectively.Libraries were sequenced on the Illumina Nextseq500 system (Illumina) and 75 bp single-end reads were generated. A minimum of 35 million and 10 million fragments per sample were analysed for RNASeq and miRNASeq respectively.

### Quality control: adapter sequence removal and low-quality sequence trimming

All raw datasets produced by deep sequencing from each library were subjected to further quality control processing, including removal of low-quality reads, 5′- and 3′-adapter contaminants and poly (A) tails. Low quality sequences (quality score threshold Q < 35) at 3′ and 5′ were then trimmed from both ends of the clean reads.

Quality control steps of mRNAseq and miRNAseq removed ~ 1–10% and ~ 6–20% of the reads, respectively. In the case of miRNAseq, many reads were discarded because having a too short insert.

The quality controls steps for mRNA sequencing data were computed with the software programs scythe (version 0.994beta; https://github.com/vsbuffalo/scythe) and sickle (https://github.com/najoshi/sickle).

The quality controls steps for miRNA sequencing data were performed using the computer program Cutadapt version 3.3 (https://cutadapt.readthedocs.io/en/stable/; https://doi.org/10.14806/ej.17.1.200). Software was run with default parameters.

### Transcript assembly and quantification

Reads derived from mRNAs and miRNAs were processed separately.

Raw reads (mRNAs and miRNAs) from all samples were aligned to the draft *S. stercoralis* genome sequence (WormBase ParaSite, version WBPS14) and then assembled into potential transcripts including the 3′ and 5′UTRs.

The *S. stercoralis* reference genome sequence and annotations were arranged to prepare the specific index files required by the different computer programs used.

The *S. stercoralis* reference genome sequence and annotations are available at the WormBase ParaSite (https://ftp.ebi.ac.uk/pub/databases/wormbase/parasite/releases/WBPS14/species/strongyloides_stercoralis/PRJEB528/strongyloides_stercoralis.PRJEB528.WBPS14.genomic.fa.gz).

Alignments were done using HISAT version 2.2.1 (https://daehwankimlab.github.io/hisat2/) and SAMtools version 1.9 (http://samtools.sourceforge.net/).

Since we aimed to investigate possible mRNA-miRNA interactions and the genome annotations did not report any information regarding the 3′UTR of mRNAs, we assembled the reads into transcripts to reconstruct their 3′UTRs.

The transcript was assembled combining the alignment data of each sample using the computer program StringTie version v2.2.0 (https://ccb.jhu.edu/software/stringtie/).

Transcript quantification (mRNAs) was performed using a selective alignment with a decoy-aware transcriptome (*S. stercoralis* reference genome and transcriptome from WormBase ParaSite) in order to mitigate potential spurious mapping of reads that actually arise from some unannotated genomic locus that is sequence-similar to an annotated transcriptome. The quantification step (mRNAs and miRNAs) estimated the expression values of each reference transcript as the relative abundance of units of Transcripts Per Million (TPM) and number of reads mapping to each transcript that was quantified.

Transcript quantification was performed using the computer program Salmon version v.1.7.0 (https://salmon.readthedocs.io/en/latest/index.html) with default settings.

The Salmon output (number of reads mapping to each transcript) was then processed by DESeq2 package of R software to identify modulated transcripts/genes.

### Functional annotation

The emapper-2.0.1 tool^44,45^, that uses precomputed orthologous groups and phylogenies from the eggNOG database to transfer functional information from fine-grained orthologs, was used in order to obtain a functional annotation. The aminoacidic sequences of the *S. stercoralis* proteins (ver. WBPS14), available at parasite.wormbase.org, were used as an input file. In this way, the genes were classified in 24 eggNOG sub-categories, with the 4 main categories being: information storage and processing, cellular processes and signaling, metabolism, and poorly characterized.

### miRNA sequencing and quantification

The analysis of miRNA went through multi-steps procedure including a preprocessing of data, detection of known and unknown miRNAs, and quantification and expression profiling. All these steps were done using the different modules of the computer program MiRDeep2 (https://github.com/rajewsky-lab/mirdeep2).

Only cleaned sequences (see “Quality control: adapter sequence removal and low-quality sequence trimming” section) that aligned to the *S. stercoralis* genome sequences (WormBase ParaSite, version WBPS14) were retrieved for the *S. stercoralis* miRNA sequences identification.

The miRNA identification process and the following estimation of the expression levels were carried out in two steps: first we identified the already known miRNAs (miRBase release 22.1—the microRNA database; https://www.mirbase.org/) and then, we identified sequences of miRNA not yet described. For the known miRNAs, as the miRNA database does not contain *S. stercoralis* miRNAs, we chose the miRNA sequences of the phylogenetically close *S. ratti*.

Expression levels of each (known or unknown) miRNA were estimated by counting the number of sequences (in each sample) mapping on the miRNA genome region.

In order to assess the number of miRNA sequences shared across *S. stercoralis*, *S. ratti*, *S. papillosus* and *C. elegans,* we compared mature *S. stercoralis* miRNA sequences obtained from sequencing with *S. ratti* and *C. elegans* miRNAs available in miRbase, and *S. papillosus* miRNAs available in the literature^18^. The miRNA sequences were considered as shared among different organisms when they showed 100% sequence identity. The Venn diagram reported in Fig. 1A was generated with the VennDiagram R package version 1.7.3.

### Differential gene expression analyses

Gene Transcripts and miRNAs were analysed separately. The analyses to identify either modulated transcripts or modulated miRNAs were conducted considering the link between a L1 and its corresponding iL3 (paired data) and Log2fold change was calculated as iL3/L1 ratio. Analyses were done using the DESeq2 library of the computer package R (version 4.0.3).

### Gene set enrichment analysis (GSEA)

The GSEA is a computational method that determines whether an a priori defined set of genes shows statistically significant, concordant differences between two biological states. GSEA v4.0.3 was used in this study to determine whether *S. stercoralis* gene sets showed biased expression in L1 and iL3. For this analysis we considered 28 gene sets: 24 gene sets were generated from the 24 eggNOG sub-categories, while 4 gene sets were manually generated to include genes known to be differentially expressed from the literature^12^, i.e., a heat-shock protein encoding gene set, a putative antigen gene set, a prokaryotes heat-shock protein gene set, and a gene set containing genes encoding products known to be immunoreactive in *S. stercoralis*-infected humans. Additionally, four randomly generated gene sets were included in the analysis to test the method robustness. The GSEA v4.0.3 software^46,47^ was used with the following parameters: scoring_scheme weighted, norm meandiv, mode Max_probe, set_max 1500, plot_top_x 20, nperm 1000, order descending, set_min 15, metric Signal2Noise, permute gene_set. P-values were adjusted performing 1,000 permutations on gene sets; threshold for statistical significance was set at p-value < 0.01 after adjusting.

### miRNA expression by RT-qPCR

To verify the expression of identified miRNAs by RNAseq in L1 and iL3, we investigated these by RT-qPCR. cDNA synthesis was performed by TaqMan Advanced miRNA Assays (ThermoFisher Scientific) from 10 ng of total RNA, according to manufacturer’ instructions. We used customized commercial TaqMan Advanced probes (ThermoFisher Scientific) (sequences are not available as part of intellectual property rights). miRNA expression analysis was made using TaqMan Fast Advanced Master Mix (ThermoFisher Scientific) according to the manufacturer’ instructions. The real time amplifications included 20 s at 95 °C, followed by 40 cycles at 95 °C for 30 s, and at 60 °C for 30 s. Thermocycling and signal detection were performed with CFX-96 Touch Real-Time PCR Detection System (Biorad). Negative controls no RT and no template control (NTC) were used. Synthetic spike-in ath-miR159a was used as internal control of reaction. The expression levels were calculated for each sample group after normalization against three reference genes STR-miR-8366A-5p, STR-miR-92-3p and STR-miR-7880L-3p (Supplementary_2) using the NRQ method^48^.

### mRNA 3′-UTR and miRNA target prediction

The tool exUTR v0.1.0^49^ was used to predict three prime untranslated regions (3′-UTR) starting from StringTie assembled mRNA sequences in fasta format. Briefly, the workflow of the software was the following: transcripts went through an open reading frame (ORF) prediction step, ORFs were then validated by aligning them to Uniprot database (https://www.uniprot.org/) using BLASTP, filtered, and self-BLASTed against transcripts sequences to determine the stop codon in transcripts; finally, 3′-UTRs sequences were extracted.

Differentially expressed miRNA sequences and predicted 3’-UTR sequences were used as input for PITA version 6^50^ in order to perform the miRNA target prediction analysis. Results were then filtered considering seeds of size 7–8 with no mismatches and having ddG ≤ − 10.

### Data analysis

Statistical analyses and graphical representations were performed using Graph Pad Prism v.8 software and R Statistical Software v.4.0.3. Multiple one-tail unpaired t-test analysis was used for the differential expression of miRNAs with qRT-PCR. A p-value < 0.05 was statistically significant.

### Ethics declarations

The study protocol received ethical clearance from the competent Ethics Committee (Comitato Etico for Clinical Research of Verona and Rovigo Provinces, no. 8634/2019). All included patients signed an informed consent form for the donation of their biological samples for research purposes. All experiments were performed in accordance with relevant guidelines and regulations.

## Supplementary Information

Supplementary Information 1.

Supplementary Information 2.

Supplementary Information 3.

Supplementary Information 4.

Supplementary Information 5.

Supplementary Information 6.

Supplementary Information 7.

Supplementary Information 8.

Supplementary Information 9.

Supplementary Information 10.

## Data Availability

All data generated or analysed during this study are included in article and its supplementary information files. Sequences have been submitted to the ENA database with project number PRJEB48672. The assembled data and scripts from this study can be requested from the corresponding author.

## References

[1] Buonfrate D (2020). The global prevalence of *Strongyloides stercoralis* infection. Pathogen (Basel, Switzerland)..

[2] McDonald HH, Moore M (2017). *Strongyloides stercoralis* hyperinfection. N. Engl. J. Med..

[3] Schär F (2013). *Strongyloides stercoralis*: Global distribution and risk factors. PLoS Negl. Trop. Dis..

[4] Nutman TB (2017). Human infection with *Strongyloides stercoralis* and other related Strongyloides species. Parasitology.

[5] Greaves D, Coggle S, Pollard C, Aliyu SH, Moore EM (2013). *Strongyloides stercoralis* infection. BMJ.

[6] Viney, M. E. & Lok, J. B. *The biology of Strongyloides spp. WormBook.* 10.1895/wormbook.1.141.2 PMC5402216 26183912

[7] Stoltzfus JD, Minot S, Berriman M, Nolan TJ, Lok JB (2012). RNAseq analysis of the parasitic nematode *Strongyloides stercoralis* reveals divergent regulation of canonical dauer pathways. PLoS Negl. Trop. Dis..

[8] Stoltzfus JD, Bart SM, Lok JB (2014). cGMP and NHR signaling co-regulate expression of insulin-like peptides and developmental activation of infective larvae in *Strongyloides stercoralis*. PLoS Pathog..

[9] Gonzalez Akimori D (2021). Transcriptional profiles in *Strongyloides stercoralis* males reveal deviations from the Caenorhabditis sex determination model. Sci. Rep..

[10] Moreira LA (2019). When a bacterium fights arboviruses. Comptes Rendus Biol..

[11] Lu MR, Lai C-K, Liao B-Y, Tsai IJ (2020). Comparative transcriptomics across nematode life cycles reveal gene expression conservation and correlated evolution in adjacent developmental stages. Genome Biol. Evol..

[12] Ramanathan R (2011). Microarray-based analysis of differential gene expression between infective and noninfective larvae of *Strongyloides stercoralis*. PLoS Negl. Trop. Dis..

[13] Marcilla A (2012). The transcriptome analysis of *Strongyloides stercoralis* L3i larvae reveals targets for intervention in a neglected disease. PLoS Negl. Trop. Dis..

[14] Rodpai R (2021). Exposure to dexamethasone modifies transcriptomic responses of free-living stages of *Strongyloides stercoralis*. PLoS One.

[15] Sarkies P (2015). Ancient and novel small RNA pathways compensate for the loss of piRNAs in multiple independent nematode lineages. PLoS Biol..

[16] Britton C, Laing R, Devaney E (2020). Small RNAs in parasitic nematodes—Forms and functions. Parasitology.

[17] Ahmed R (2013). Conserved miRNAs are candidate post-transcriptional regulators of developmental arrest in free-living and parasitic nematodes. Genome Biol. Evol..

[18] Holz A, Streit A (2017). Gain and loss of small RNA classes-characterization of small RNAs in the parasitic nematode family Strongyloididae. Genome Biol. Evol..

[19] Wienholds E, Plasterk RHA (2005). MicroRNA function in animal development. FEBS Lett..

[20] DeVeale B, Swindlehurst-Chan J, Blelloch R (2021). The roles of microRNAs in mouse development. Nat. Rev. Genet..

[21] Karp X, Hammell M, Ow MC, Ambros V (2011). Effect of life history on microRNA expression during *C. elegans* development. RNA.

[22] Karp X, Ambros V (2012). Dauer larva quiescence alters the circuitry of microRNA pathways regulating cell fate progression in *C. elegans*. Development.

[23] de Lencastre A (2010). MicroRNAs both promote and antagonize longevity in *C. elegans*. Curr. Biol..

[24] Bethke A, Fielenbach N, Wang Z, Mangelsdorf DJ, Antebi A (2009). Nuclear hormone receptor regulation of microRNAs controls developmental progression. Science.

[25] Hammell CM, Karp X, Ambros V (2009). A feedback circuit involving let-7-family miRNAs and DAF-12 integrates environmental signals and developmental timing in *Caenorhabditis elegans*. Proc. Natl. Acad. Sci. U. S. A..

[26] Starega-Roslan J, Koscianska E, Kozlowski P, Krzyzosiak WJ (2011). The role of the precursor structure in the biogenesis of microRNA. Cell. Mol. Life Sci..

[27] Pomari E (2017). Clinical impact of miR-223 expression in pediatric T-cell lymphoblastic lymphoma. Oncotarget.

[28] Bortoluzzi S, Lovisa F, Gaffo E, Mussolin L (2017). Small RNAs in circulating exosomes of cancer patients: A minireview. High-throughput.

[29] Abbott AL (2011). Uncovering new functions for microRNAs in *Caenorhabditis elegans*. Curr. Biol..

[30] Bartel DP (2009). MicroRNAs: Target recognition and regulatory functions. Cell.

[31] McKerrow JH (1990). *Strongyloides stercoralis*: Identification of a protease that facilitates penetration of skin by the infective larvae. Exp. Parasitol..

[32] Lackey A (1989). Extracellular proteases of Onchocerca. Exp. Parasitol..

[33] Hunt VL, Tsai IJ, Selkirk ME, Viney M (2017). The genome of Strongyloides spp. gives insights into protein families with a putative role in nematode parasitism. Parasitology.

[34] Varatharajalu R, Parandaman V, Ndao M, Andersen JF, Neva FA (2011). *Strongyloides stercoralis* excretory/secretory protein strongylastacin specifically recognized by IgE antibodies in infected human sera. Microbiol. Immunol..

[35] Hunt VL (2016). The genomic basis of parasitism in the Strongyloides clade of nematodes. Nat. Genet..

[36] Hunt VL, Hino A, Yoshida A, Kikuchi T (2018). Comparative transcriptomics gives insights into the evolution of parasitism in *Strongyloides nematodes* at the genus, subclade and species level. Sci. Rep..

[37] Baskaran P, Jaleta TG, Streit A, Rödelsperger C (2017). Duplications and positive selection drive the evolution of parasitism-associated gene families in the nematode *Strongyloides papillosus*. Genome Biol. Evol..

[38] Britton C, Winter AD, Gillan V, Devaney E (2014). microRNAs of parasitic helminths—Identification, characterization and potential as drug targets. Int. J. Parasitol. Drugs Resist..

[39] Li C (2008). An essential role for DYF-11/MIP-T3 in assembling functional intraflagellar transport complexes. PLoS Genet..

[40] Hong RL (2019). Evolution of neuronal anatomy and circuitry in two highly divergent nematode species. Elife.

[41] Ashton FT, Bhopale VM, Fine AE, Schad GA (1995). Sensory neuroanatomy of a skin-penetrating nematode parasite: *Strongyloides stercoralis*. I. Amphidial neurons. J. Comp. Neurol..

[42] Lopez PM, Boston R, Ashton FT, Schad GA (2000). The neurons of class ALD mediate thermotaxis in the parasitic nematode, *Strongyloides stercoralis*. Int. J. Parasitol..

[43] Wolfgang, K. & Donald, F. *Changes in Beta-Oxidation and Related Enzymes During the Life Cycle of Strongyloides ratti (Nematoda)*. (The Journal of Parasitology, 1971). 4400482

[44] Huerta-Cepas J (2017). Fast genome-wide functional annotation through orthology assignment by eggNOG-Mapper. Mol. Biol. Evol..

[45] Huerta-Cepas J (2019). eggNOG 5.0: A hierarchical, functionally and phylogenetically annotated orthology resource based on 5090 organisms and 2502 viruses. Nucleic Acids Res..

[46] Subramanian A (2005). Gene set enrichment analysis: A knowledge-based approach for interpreting genome-wide expression profiles. Proc. Natl. Acad. Sci. U. S. A..

[47] Mootha VK (2003). PGC-1alpha-responsive genes involved in oxidative phosphorylation are coordinately downregulated in human diabetes. Nat. Genet..

[48] Hellemans J, Mortier G, De Paepe A, Speleman F, Vandesompele J (2007). qBase relative quantification framework and software for management and automated analysis of real-time quantitative PCR data. Genome Biol..

[49] Huang Z, Teeling EC (2017). ExUTR: A novel pipeline for large-scale prediction of 3′-UTR sequences from NGS data. BMC Genom..

[50] Kertesz M, Iovino N, Unnerstall U, Gaul U, Segal E (2007). The role of site accessibility in microRNA target recognition. Nat. Genet..

