# Supplementary figures and images for: Identification of miRNAs of Strongyloides stercoralis L1 and iL3 larvae isolated from human stool
Source: Sci Rep. 2022 Jun 15;12:9957. doi: 10.1038/s41598-022-14185-y (PMC9200769; doi:10.1038/s41598-022-14185-y)

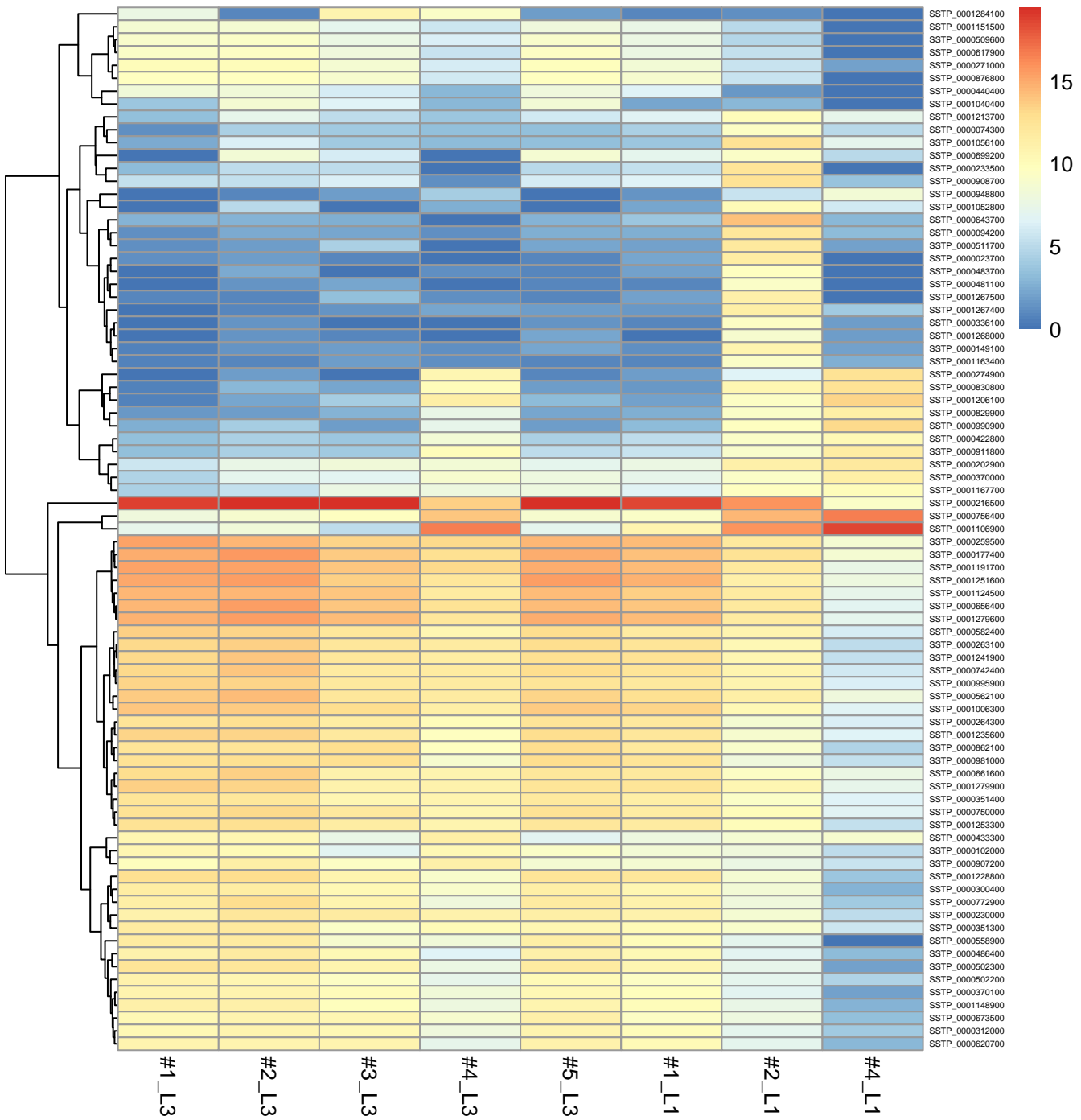

Supplement: Supplementary file 1 — Supplementary Information 1. [file 41598_2022_14185_MOESM1_ESM.pdf]

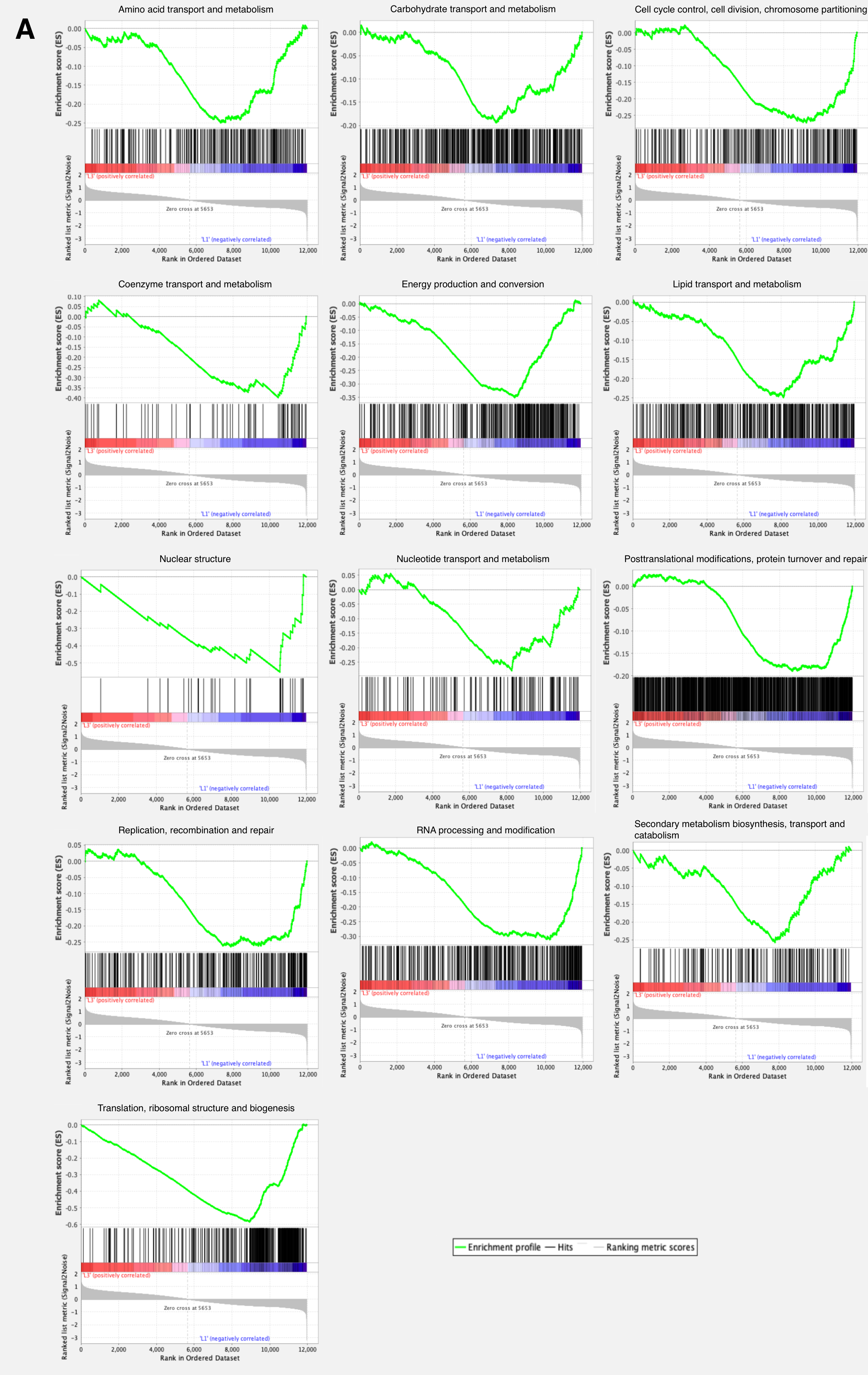

Supplement: Supplementary file 2 — Supplementary Information 2. [file 41598_2022_14185_MOESM2_ESM.png]

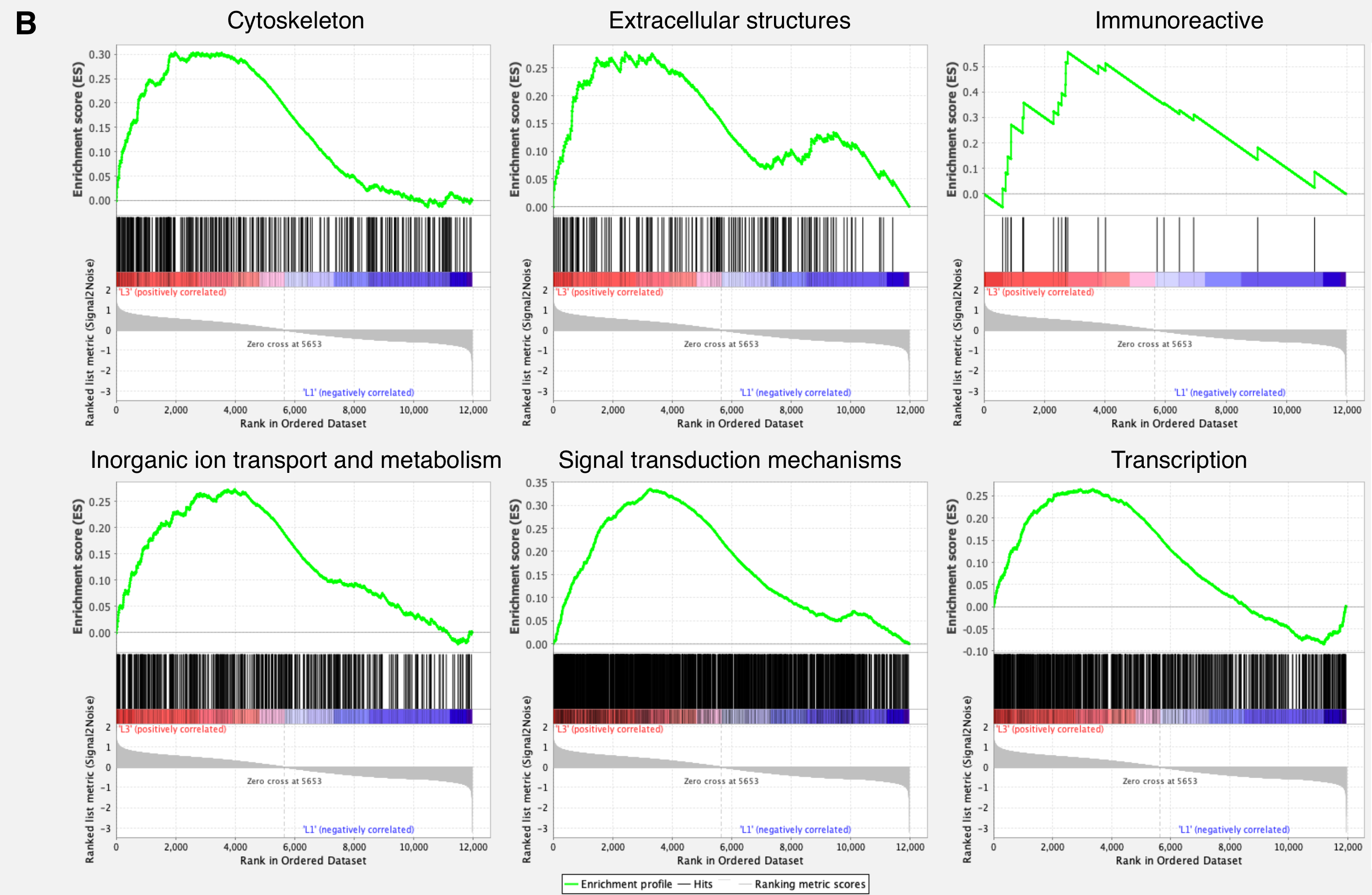

Supplement: Supplementary file 3 — Supplementary Information 3. [file 41598_2022_14185_MOESM3_ESM.png]
