# Supplementary material for: Identification of miRNAs of Strongyloides stercoralis L1 and iL3 larvae isolated from human stool
Source: Sci Rep. 2022 Jun 15;12:9957. doi: 10.1038/s41598-022-14185-y (PMC9200769; doi:10.1038/s41598-022-14185-y)
Supplement: Supplementary file 4 — Supplementary Information 4. [file 41598_2022_14185_MOESM4_ESM.docx]

**Supplementary information**

**Figure S1.** Heatmap of 8 samples using the 81 differentially expressed genes between L1 and iL3 stages of *S. stercoralis*. The heatmap function calculated a clustering based on the euclidean distances between the rows/columns of the distance matrix (gene expression levels as normalized gene counts). Data were obtained from five patients (#1-5). A total of 8 samples were analysed.

The names of transcripts are reported according to the Wormbase annotations (WormBase ParaSite, version WBPS14).

**Figure S2A_B.** Gene set enrichment analysis plots. Enrichment plots of significantly enriched gene-sets for L1 (A) and iL3 (B). The GSEA tool generates a ranked list of genes based on the signal-to-noise ratio and calculates the enrichment score (ES), which reflects the degree to which a gene set is overrepresented at the top or bottom of the ranked list of genes. The top portion of the plot shows the running ES for the gene set as the analysis walks down the ranked list; the middle portion of the plot shows where the members of the gene set appear in the ranked list of genes; while the bottom portion of the plot shows the value of the ranking metric as you move down the list of ranked genes.

**Supplementary materials**

**Supplementary material_1.** Name and sequence of miRNAs.

**Supplementary material_2.** Differential analysis of miRNAs.

**Supplementary material_3.** Differential analysis of long transcripts.

**Supplementary material_4.** Data set from Gene set enrichment analysis.

**Supplementary material_5.** Data set from the targeting analysis between miRNAs and mRNAs.

**Supplementary material_6.** Mapping positions of miRNAs on *S. stercoralis* genome version WBPS14.
