# Supplementary material for: Identification of miRNAs of Strongyloides stercoralis L1 and iL3 larvae isolated from human stool
Source: Sci Rep. 2022 Jun 15;12:9957. doi: 10.1038/s41598-022-14185-y (PMC9200769; doi:10.1038/s41598-022-14185-y)
Supplement: Supplementary file 5 — Supplementary Information 5. [file 41598_2022_14185_MOESM5_ESM.docx]

**MATURE miRNA sequence**

>STR-MIR-1-5P

CATGCTTCTTTATAGTGCCATA

>STR-MIR-1-3P

TGGAATGTAAAGAAGTATGCA

>STR-MIR-7880A-5P

CAGTTGGATCGATCAGCAAAAA

>STR-MIR-7880A-3P

TTTGCGACTGTATCCAAACTACG

>STR-MIR-50-5P

TGATATGTCTGGTATTCTTGGGT

>STR-MIR-50-3P

CGAGTAATATTAGACATATCGAG

>STR-MIR-8365-5P

CACTCCTTTTCGTTTGCTAATA

>STR-MIR-8365-3P

ATAGGAAATGAATAAGGAGAAT

>STR-MIR-7880B-1-5P

TGGCTTGGATCTTTCTGCAAACA

>STR-MIR-7880B-3P

TTTGCGACCGAATCCAGGCCACG

>STR-MIR-8366A-5P

ACAGTATTGACTAACGTGTATG

>STR-MIR-8366A-3P

TACCCGTAGTCCAATGCTGTCA

>STR-MIR-7880B-2-5P

TGGTTGGATCGATCTGCAAACA

>STR-MIR-7880B-3-5P

TGGTTGGATCAGTTTGCAAACA

>STR-MIR-7880B-4-5P

TGGTTGGATCGATCAGCAAATA

>STR-MIR-7880C-5P

TGGCTGGAGTTGGAGCAAATG

>STR-MIR-7880C-3P

TTTGCGACCGAATCCAAGCCTGA

>STR-BANTAM-5P

TGTGATTTCAAATGGACTCCAGA

>STR-BANTAM-3P

TGAGATCATTCTGAAATCACGAT

>STR-MIR-7880D-5P

GATTGGGTCAGGCAGCAAATA

>STR-MIR-7880D-3P

TTTGCGACTGTATCCACATCACT

>STR-MIR-71-5P

TGAAAGACATGGGTAGTGAGACG

>STR-MIR-71-3P

TATCACTATTCTGTTTTTCTCC

>STR-MIR-7880E-5P

TGGACTGGATAGTTGCAAACA

>STR-MIR-7880E-3P

TTTGCGACCAAATCCAGGCCACG

>STR-MIR-7880F-5P

GGCCTGGTTAGTCGCAAAGA

>STR-MIR-7880F-3P

TTTGCGATTGTTTCCAGGCCAGC

>STR-MIR-7880G-5P

GGTCAGATGAGGTCTGCAAATT

>STR-MIR-7880G-3P

TTTGCGACTAAATTTGGACCAA

>STR-MIR-252A-5P

TTAAGTAGTTGTGCCGTAGGTCG

>STR-MIR-252A-3P

ACCTACACACGGCTCCTTAAGA

>STR-MIR-7880H-5P

GGTTAGGATCAGTTTGGCAAATA

>STR-MIR-7880H-3P

TTTGCGACTGTATCCAAAACCCA

>STR-MIR-7880I-5P

GGTCTAGTGATGGTAGCAAATT

>STR-MIR-7880I-3P

TTTGCGACCAATCCAGGACCCG

>STR-MIR-81A-5P

ATGGGTCTATATGATTCTCATG

>STR-MIR-81A-3P

TGAGATCATGTTAGACTCATAT

>STR-MIR-8367-5P

GAGTCAAGTAATCTTGGTTAAA

>STR-MIR-8367-3P

TAACCGGATATATTTGACTACAG

>STR-MIR-7880E-2-5P

TGGTTGGAATGGTCAGCAAATA

>STR-LIN-4-5P

TCCCTGAGACTCTACTTGTGA

>STR-LIN-4-3P

CCAAGTTAGATGATCAGGTACC

>STR-MIR-8368-5P

TCCCTGAGATTTCATATAACCGG

>STR-MIR-8368-3P

CGTATATGAATGATCAGGTACC

>STR-MIR-236-5P

CGTCATTATCGCAGCTATTAGA

>STR-MIR-236-3P

TAATACTGTCAGGTAATGACGCT

>STR-MIR-81B-5P

ATGGGCCTATGTGTGTCTCGTG

>STR-MIR-81B-3P

TGAGATCATATTAGACCCATCT

>STR-MIR-81C-5P

ATAGGTCTATTTGCTTTCTCATG

>STR-MIR-81C-3P

TGAGATCAAATTAGACTTATCG

>STR-MIR-72-5P

AGGCAAGATGTTGGCATAGCTGA

>STR-MIR-72-3P

AGTTATGCTATATGTTGCCAGA

>STR-MIR-8369-5P

CTGGAGCCCAGGTTGACAATGC

>STR-MIR-8369-3P

TTTGGCACCATGGGACTCTAAT

>STR-MIR-7880J-5P

TGGTCGGAATAGTTGCAAATT

>STR-MIR-7880J-3P

TTTGCGACCTATTTCCAGGCTACT

>STR-MIR-5359-5P

TCTGGGATTGTTTATCTTGGTA

>STR-MIR-5359-3P

CAAGAATAAACATTCCTTGAC

>STR-MIR-86-5P

TAAGTGAATGCTTTGCCACAGTCT

>STR-MIR-86-3P

CCTGCTGCAACATTCACTTGGC

>STR-MIR-92-5P

AGGTTGGAGCATGCGCCATATT

>STR-MIR-92-3P

TATTGCACACGTCCCGGCCTGA

>STR-MIR-7880K-5P

GGTCAGAAATGGTTGCAAATTAGTAG

>STR-MIR-7880K-3P

TTTGTGACCTTTCAATGATCAG

>STR-MIR-8370-5P

AGATGCAGGTTGGGTCTTTATG

>STR-MIR-8370-3P

TAAGGCACAACGTTGGCATCTCT

>STR-MIR-7880L-5P

GGCTTAGGATGGTGCAAATT

>STR-MIR-7880L-3P

TTTGCGACCATATCCAAAGCCCGT

>STR-MIR-7880M-5P

GGTCGGTAGAAGAAGCAAATT

>STR-MIR-7880M-3P

TTTGCGCTCGATACCTGACCAC

>STR-MIR-8371-5P

AGATGGGTGAGTTTGCCAAATT

>STR-MIR-8371-3P

TTTGCGAACGACCCTGTCCAA

>STR-MIR-7880N-5P

TGATGGATAAGGAGCAAATT

>STR-MIR-7880N-3P

TTTGCAACTTTATCCATTTCACT

>STR-MIR-252B-5P

TTAAGTAGTAGTGCCGCAGGTTG

>STR-MIR-252B-3P

ACCTTCACACCACTGCTTAAT

>STR-MIR-7880O-5P

TGGTTGGATGAGAAGCAAATT

>STR-MIR-7880O-3P

TTTGCGACTATATCCAGGCCATT

>STR-MIR-7880P-5P

GGCCAGTGATGTCTCAAGTT

>STR-MIR-7880P-3P

TTTGGGACCATATCTGTGCCAG

>STR-MIR-1175-5P

CGGTGAAGAAGAGAATCTGACT

>STR-MIR-1175-3P

TGAGATTCTCTTTTCTTCATTC

>STR-MIR-7880Q-5P

GGCTGGTTAGGCTGCAAATC

>STR-MIR-7880Q-3P

TTTGCGACCAAAATCATGCCAC

>STR-MIR-8372-5P

ACGTTTGATCGAGCTTTAAATA

>STR-MIR-8372-3P

TTAAAGCTTAGATTAAACGTCT

>STR-MIR-8373-5P

AGAATAACTATATATTATCACCAGTC

>STR-MIR-8373-3P

TGATATGTACAGTTATTCTGT

>STR-MIR-8366B-5P

ACAGTATTGATATGTGGGTGAC

>STR-MIR-8366B-3P

TACCCGTAATCCAATGCTGCAA

>STR-MIR-7880R-5P

TGGCTGGGTTGCTCTGCAAAAA

>STR-MIR-7880R-3P

TTTGCGACCAATCCAAGCCAAA

>STR-MIR-7880S-5P

TGGCTGGAAAGGCTGCAAATT

>STR-MIR-7880S-3P

TTTGCGACCTATATCCAGGCCACG

>STR-MIR-8374-5P

CAACATTCAATTCATATAAAACAATTT

>STR-MIR-8374-3P

TAAATATGAATGTGAATGTTCT

>STR-MIR-8375-5P

TTTTAAGCATCGGTAACAAACA

>STR-MIR-8375-3P

TTTGGGACCTGTGTTTAAGTAA

>STR-MIR-8376-5P

TACTCAGTACGTAACAAAGATT

>STR-MIR-8376-3P

TTTGCAACGTATCTGGGTTTAC

>STR-MIR-7880T-5P

GCACTTGGTTGGAAGCAAATA

>STR-MIR-7880T-3P

TTTGCGACCAAATCATGTGCAT

>STR-MIR-9-5P

TCTTTGGTTATTTAGCTATATGA

>STR-MIR-9-3P

TAAGCTGAACGATCAAAGGAA

>STR-MIR-8377-5P

TTTAACGCGGATAGATGAATT

>STR-MIR-8377-3P

TTCATTGTCCAGCGTTGAAC

>STR-MIR-87A-5P

CATCGGAGACTTTTGGACTCAACCT

>STR-MIR-87A-3P

GTGAGCAAGAGCTTCCGGTGTG

>STR-MIR-47-5P

TAGGAGAGCTGTCTTTGTCGGT

>STR-MIR-47-3P

TGTCATGGAGTAGCTCTCTTAGA

>STR-MIR-7880T-5P

TGCCCCAGTAAGGCCACAAAT

>STR-MIR-7880T-3P

TTTGCGACCATTCCAGGGCTA

>STR-MIR-8378-5P

CATTCTGCATTGCTTAATAACT

>STR-MIR-8378-3P

TATTATGCAAAGTAGAATGTA

>STR-MIR-84-5P

TGAGGTAGTGTTAAATATTGTT

>STR-MIR-84-3P

CAATATTTTATTCTACTTCATC

>STR-MIR-8379A-5P

AGCTGTCTTTTACAGTATCAGA

>STR-MIR-8379A-3P

TAATACTGCCTTAGACGGCTAA

>STR-MIR-76-5P

CGAGTTTCGTGATGTCGAATA

>STR-MIR-76-3P

TTCGTTGTTACTGAAACTCGTC

>STR-MIR-8380-5P

TGGGTATGTTTTTCGTCCAAGA

>STR-MIR-8380-3P

TTGCACGATTTACATACTCACA

>STR-MIR-8381-5P

CAAAAATTCGCAATCAAAAGC

>STR-MIR-8381-3P

TTTTGTATTGCTGAATTTTTGAT

>STR-MIR-8382-5P

TGGAATTCTAAGAAGCGAAACAAGATAAT

>STR-MIR-8382-3P

TTTGGCACTTAAGAATTCCACT

>STR-MIR-7880V-5P

GGTCGGATGATTATTGCGAATT

>STR-MIR-7880V-3P

TTTGCGATAACATTCAAGACCAA

>STR-MIR-8383-5P

TCGAATCGTCTAGAAGTTAATT

>STR-MIR-8383-3P

ATTGGCTTCGTGATGATTCAAA

>STR-MIR-7880W-5P

GACAGGAATTCTAATTGCAAATT

>STR-MIR-7880W-3P

TTTGCAAAGTATATCCAAGTCGA

>STR-MIR-8384-5P

ATGGATCTTTGTATGTCTCATG

>STR-MIR-8384-3P

TGAGATTATGAAGATTCATTA

>STR-MIR-8386-5P

CGGGTTTGTTACTTGTTGAATT

>STR-MIR-8386-3P

TTCACCGGGTAGACAAACGCGCG

>STR-MIR-8387-5P

AAATTTCATGATTCGGTAGGA

>STR-MIR-8387-3P

TCACCGAAGTCATGAAGTTTC

>STR-MIR-40-5P

AGAGTTTGTCGGCATGGTGACT

>STR-MIR-40-3P

TCACCGGGTGTACGAATTCTCA

>STR-MIR-124-5P

CGCTTTCATCCGTGACTTTAGA

>STR-MIR-124-3P

TAAGGCACGCGGTGAATGC

>STR-MIR-184-5P

ACTTATCAACTTCATGCCTTGT

>STR-MIR-184-3P

TGGACGGAAGTTTGATAAGAGA

>STR-MIR-8388-5P

AAGCAAAATGGGATGCAAATA

>STR-MIR-8388-3P

TTTGAGACCTCATCTTAGCTTAG

>STR-MIR-8389-5P

AATATCTGAATTGTTTACAAAAC

>STR-MIR-8389-3P

CATGTAAACATTTTAGATATACA

>STR-MIR-240-5P

AGAAGTTTGGTAGTCATGAACA

>STR-MIR-240-3P

TACTGGCCTTCAAACTTCTTA

>STR-MIR-8390-5P

TATTTCAAAAGTTGCATATGACA

>STR-MIR-8390-3P

TAAATGCATCTTTTGAGATAGA

>STR-MIR-8391-5P

TTGTGGAGTCGTGGAAAAATG

>STR-MIR-8391-3P

TCAATCCTCGTACTTCACAACA

>STR-MIR-8392-5P

AACTCTTGATTTCTGTAGAAT

>STR-MIR-8392-3P

TTCACCGGAAACCAGGAGAGG

>STR-MIR-37A-5P

AGTGAATGTTACTTCGGTAATG

>STR-MIR-37A-3P

TCACCGGGTATACATTCATCAA

>STR-MIR-255-5P

GTAAGAAGTCAAATCGGTTTC

>STR-MIR-255-3P

AAACTGAATTGATTCCTTACAG

>STR-MIR-8393-5P

AGCATTTGAATCATCGATGATT

>STR-MIR-8393-3P

TCACCGGGATATCAGATGCTC

>STR-MIR-360-5P

TTGTGACCGTTGTAACGGCTATTC

>STR-MIR-360-3P

ATGTCGGATACAATGAGCACAAA

>STR-MIR-8394-5P

AAGGTGTGGTCGTCGCCATTATA

>STR-MIR-8394-3P

TATGGCACGTACACCATCTTC

>STR-MIR-58-5P

ATGACACTGAATGAACTGACG

>STR-MIR-58-3P

TGAGATCATATTAGTGTCATTT

>STR-MIR-37B-5P

AAGTAATTGTTTCCATGTTGAT

>STR-MIR-37B-3P

TCACCGGGAATACAATTACCAAT

>STR-MIR-8379B-3P

TAGCCGTCTAAGGCAGTATTAGA

>STR-MIR-8379B-5P

TGATACTGTAAAAGACAGCTAA

>STR-MIR-279-5P

CAAGGTAATTTCATAGTCATG

>STR-MIR-279-3P

TGACTAGAACATTACTTTG

>STR-MIR-87B-5P

CGTTAAACATTGGCTCAACA

>STR-MIR-87B-3P

TTGAGCAATGTTTTAATGCA

>STR-MIR-2-5P

TTCAAAAGACTGTGTTTCAGGA

>STR-MIR-2-3P

TATCACAGTTATTTTGAATCTG

>STR-MIR-234-5P

ATAGGTATTCTCAAACAATTGCATTA

>STR-MIR-234-3P

TTATTGCTTGAGAATGCACAT

>STR-MIR-7880X-5P

TGGCCTGGATTCGGTCGCAAAGA

>STR-MIR-7880X-3P

TTTGCTGATCGATCCAACCATG

>STR-MIR-8395-5P

ACGACCTTATTTTGTCTTCAA

>STR-MIR-8395-3P

TAAGGCATTTTAAGGTTGTTC

>STR-MIR-8396-5P

CGGGTTTATCATTGCCAAACT

>STR-MIR-8396-3P

TTTGGCACTGTATAGGCTCCAA

>STR-MIR-34A-5P

TGGCAGTGTGGTTAGCTGGTTG

>STR-MIR-34A-3P

ACAGCTCACTCAACTGCCAAG

>STR-MIR-34B-5P

TGGCAGTGTAGTTAGCTGGTTG

>STR-MIR-34B-3P

ACAGCTTACCAAATTGCCATC

>STR-MIR-8397-5P

AGGTGTAGATTGCCATTCAACTG

>STR-MIR-8397-3P

TGAAAGACGTTCTATATCTCT

>STR-MIR-8398-5P

AGTTCTTTCTTTTTAGCTTTAG

>STR-MIR-8398-3P

TAAGCTGCCATGAAAGAACTAA

>STR-MIR-8399-5P

TTTCAGTAGGGACTTTATTAAA

>STR-MIR-8399-3P

TAATGATGTCTTACGGAAATA

>STR-MIR-8400-5P

TGTGGGTTAAATCATCTAGATT

>STR-MIR-8400-3P

TTCTAGGTAATTTAAGCTACAAA

>STR-MIR-8401-5P

TTGAATCATCACGAAGCCAATT

>STR-MIR-8401-3P

ATTAACTTCTAGACGATTCGAAT

>STR-MIR-7880Y-5P

GGTCAGATGATGTCCACAAATT

>STR-MIR-7880Y-3P

TTTGCGACTAAATCTAGACC

>STR-MIR-8402-5P

TCAAAAATTCAGCAATACAAAAGC

>STR-MIR-8402-3P

TTTTGATTGCGAATTTTTGTAA

>STR-MIR-34C-5P

TGGCAGTGTGATTAGCTGGTTG

>STR-MIR-34C-3P

ACAGCTCATTAAACTGCCTCT

>SS--28-5P

AGATGGGTGAGTTTGTCAAATT

>SS--28-3P

TTTGCGAACGATCCTGTCCAA

>SS--29-5P

TGGTCGGGTGAGAAGCAAATT

>SS--29-3P

TTTGCGACTATATCCAGACCATT

>SS--30-5P

GGTTAGGATCAGTTTGGCAAATA

>SS--30-3P

TTTGCGACTGTATCCAAAACCCG

>SS--31-5P

TGGTTGGATCGATCAGCAAATA

>SS--31-3P

TTTGCGACCGAATCCAGGCCAC

>SS--32-5P

AATTGGTTAGGCTGCAAATC

>SS--32-3P

TTTGCGACCAAAACCAAGACA

>SS--33-5P

AGTCTAGTGATGGTAGCAAATT

>SS--33-3P

TTTGCGACCAATCCAAGACTGA

>SS--34-5P

GGTCAGATGATGTCTGCAAATT

>SS--34-3P

TTTGCGACTAAATCCAGACCAA

>SS--35-5P

TGGCTGGAGTTGGAGCAAATA

>SS--35-3P

TTTGCGACCAAATCCAAGCCTG

>SSCEL-MIR-80-3Z36-5P

GTGGTTTCAAATGAACTCCAGA

>SSCEL-MIR-80-3Z36-3P

TGAGATCATTATGAAATCACG

>SS--37-5P

GACAGGGAGTATATTTGCAAATT

>SS--37-3P

TTTGCAAAGTATACCCAAGTCGA

>SS--39-5P

ATGGATCTTTGTATGTCTCATG

>SS--39-3P

TGAGATTATAAAGATTCATTA

>SS--40-5P

GGTCAGATAAAGTCTGCAAATT

>SS--40-3P

TTTGCGACTAAATCCCGACCAA

>SS--41-5P

AGCCGGATCTTTAGCAAATT

>SS--41-3P

TTTGCAGATAGATTCCAGCCTG

>SSCEL-MIR-80-3Z42-5P

ATGAGCCTATTTGTGTCTCGTG

>SSCEL-MIR-80-3Z42-3P

TGAGATCAAACTAGACTCATT

>SSCEL-MIR-75-3Z43-5P

CTTTGGTGATTTAGCCGTAATGA

>SSCEL-MIR-75-3Z43-3P

ATAAAGCTAGGTTACCAAAGCT

>SSCEL-MIR-60-3Z44-5P

CATTCTACATTGCTTAATAACT

>SSCEL-MIR-60-3Z44-3P

TATTATGCAAAGTAGAATGC

>SSCEL-MIR-80-3Z45-5P

ATGGCACTGGATGAGCTGACG

>SSCEL-MIR-80-3Z45-3P

TGAGATCACATCAGTGTCATTT

>SSCEL-MIR-232-3Z46-5P

TGTGTCAGTACTTGCATTTACA

>SSCEL-MIR-232-3Z46-3P

TAAATGCATCTCACTAGCACAA

>SSCEL-MIR-124-3Z47-5P

AGATGCAGGTTGGTTCTTTATG

>SSCEL-MIR-124-3Z47-3P

TAAGGCACAACATTGGCATCTCT

>SS--48-5P

TACTCAGTACGTAACAAAGA

>SS--48-3P

TTTGCAACGTATCTGAGTTTAC

>SSCEL-MIR-228-5Z49-5P

CACGCGTCAATCGTAGCCATCTC

>SSCEL-MIR-228-5Z49-3P

AATGGCACCTTTGAACGTGTGC

>SS--50-5P

TGGCCGGTAAGGGCGCAAATA

>SS--50-3P

TTTACGACCTAATCCGTGTCATT

>SS--51-5P

ACTTATCAACTTCATGCCTTGT

>SS--51-3P

TGGACGGAAGTTTGATAAGTGA

>SSCEL-MIR-35-3Z52-5P

AGCATTTGAATCATCGATGAT

>SSCEL-MIR-35-3Z52-3P

TCACCGGGATATCAAATGCTA

>SSCEL-MIR-360-5Z53-5P

TTGTGACCGTTGTAACGGCTGTTC

>SSCEL-MIR-360-5Z53-3P

ATGTCGGATACAATGAGCACAAAA

>SS--54-5P

TGGCCAGTGATGTCTCAAGTT

>SS--54-3P

TTTGGGACCATAATTGTGCCAGT

>SSCEL-MIR-60-3Z55-5P

CTTTGTAGCAAGCACAATTACT

>SSCEL-MIR-60-3Z55-3P

TATTATGCCTGACTACAAAGTCT

>SS--56-5P

GATTCGATGATTACGCAAATT

>SS--56-3P

TTTGCGTATAAATCCTTGAATCTC

>SS--57-5P

AACCTTAAGAAGACGGCTAACT

>SS--57-3P

TTGTCTACTTCAAGAGGAAGA

>SS--58-5P

AACCTTAAGAAGACGGTCAACT

>SS--59-5P

GGTCGGTAGAAGAAGCAAATT

>SS--59-3P

TTTGCGCTTGGTACCTGACTAC

>SSCEL-MIR-35-3Z60-5P

AGTAATTGTTTCCATGTTGAGT

>SSCEL-MIR-35-3Z60-3P

TCACCGGGAATGCAATTACTAG

>SSCEL-MIR-790-5Z61-5P

TGGAATTCTAAGAAGCGAAACA

>SSCEL-MIR-790-5Z61-3P

TTTGGCACTTGAGAATTCCACT

>SS--62-5P

TACCTCAGGAAACGGATAAATT

>SS--62-3P

TTTACCGGTTTCCAAGAGGTCG

>SSCEL-MIR-790-5Z63-5P

AGGGTTTAACTTTGCCAAACT

>SSCEL-MIR-790-5Z63-3P

TTTGGCACAGTATAGGCTCCAA

>SS--64-5P

TGAGATATAGAAAATGATGAC

>SS--64-3P

TCATCGTTTATCATATCTCAAG

>SSCEL-MIR-234-3Z65-5P

AGGTATTCTCAAACAATTGCA

>SSCEL-MIR-234-3Z65-3P

TTATTGCTTGAGAATACATAA

>SS--66-5P

TTGTGGAGCTGTGGAAAAAGTGT

>SS--66-3P

TAAATCCTCGGCTTCACAAT

>SSCEL-MIR-87-3Z67-5P

ACGTTAAACATTGGCTCAACA

>SSCEL-MIR-87-3Z67-3P

TTGAGCAATGTCTTAATGCAA

>SS--68-5P

AGGTTGGATATTGTTATCAAATT

>SS--68-3P

TTTGGGAACAAATCCAATACCTT

>SS--69-5P

TGAGATATGGGAAATGATGACA

>SS--69-3P

TCATCGTTTATCATATCTTAAG

>SS--70-5P

AACCTCGAGAAAATAATTAAA

>SS--70-3P

TTAATGATTTTCAAGGGGGAG

>SS--72-5P

TGAGATATAAAAAATGATGACA

>SS--75-5P

TGAGATATGAAGAATGATGACA

>SS--75-3P

TCATCGTTTTTCATATCTTAAG

>SSCEL-MIR-2-3Z76-5P

TGATTCAAGGGACTGTGTTTC

>SSCEL-MIR-2-3Z76-3P

TATCACAGTTTTCTTGAATTTG

>SS--77-5P

TGTTGGATAGATCAAAAAAAAC

>SS--77-3P

TTTATTTTGATCTACCCAACAT

>SS--78-5P

TGATTTTTTTTTATTTGATAAAG

>SS--78-3P

TCAGATAAAAAAAAATCATAA

>SS--79-5P

AACCTTAAGAAGACGGCCAACT

>SS--79-3P

TTGTCTACTTCAAGGGGAAG

>SSCEL-MIR-71-5Z81-5P

AGATATTATTTGTCGGTCAAC

>SSCEL-MIR-71-5Z81-3P

TGAAAGACAAATTATATCTTT

>SS--82-3P

TCATCGTTTCTTATATCTCAAG

>SS--83-3P

TCATCGTTATCATATCTCAAG

>SS--85-3P

TTGTCTACTTCAAAAGGAAGA

>SS--86-3P

ATCGTTTGTCATATCTTAAGA

>SS--87-3P

TCATCATTTATCATATCTCA

>SS--88-5P

CTTAAGATATAAAAAATGATG

>SS--88-3P

ATCATTTCTCATATCTTAAGA

>SS--89-5P

TAAGATATAGAAAATGATGAT

>SS--89-3P

TCATCATTTCTTATATCTTAA

>SS--90-5P

TTGACCAGGTTTGAAAGAAAA

>SS--90-3P

TTCTGTCAACCCCGGTCGAA

>SS--92-5P

TCAATTTTGCTACTCTTCAAAT

>SS--92-3P

TTGAAGTATAGTAAAAGTTTGAT

>SSCEL-MIR-1820-5Z93-5P

AAAAATTCAGCAATACAAAAG

>SSCEL-MIR-1820-5Z93-3P

TTTTGATTGTGAATTTTTGT

>SS--94-3P

CTGTCTACTTCAAGAGGAAGA

>SS--95-5P

GTCGTTCAAAAATAAATGTCG

>SS--95-3P

ACATTCATTTTTGAACGACCT

>SS--96-5P

CGTGATTTCATAATGATCTCAGA

>SS--96-3P

TGGAGTTCATTTGAAACCACAAT

>SS--97-5P

TAAGTAATTATAGTAAGGAAA

>SS--97-3P

TTCCTTAATATAAAATTACT

>SS--98-5P

TTTGTAGTCAGGCATAATACTT

>SS--98-3P

TAATTGTGCTTGCTACAAAGTCT

>SS--99-3P

TTGTTTACTTCAAGAGGAAG

>SS--100-5P

AAACCTGTAGCTACGAGCTTAT

>SS--100-3P

TAAGCATCGAGTGACAGGTTAA

>SS--102-5P

TTAACCGGGTTTAAAAGAAAA

>SS--102-3P

TTTCTGTCAACCTCGGTCGAAA

>SS--103-5P

TGGCCTGGATTCGGTCGCAAAA

>SS--103-3P

TTTGCAGATCGATCCAACCACG

>SS--104-5P

TAAATTGAAGATGGAGTATCT

>SS--104-3P

TACTTCACTTGTCAATTTAATT

>SS--105-5P

GGTCTGGATTTAGTCGCAAATT

>SS--105-3P

TTTGCAGACATCATCTGACCAA

>SS--106-5P

TGGCCTGGATTCGGTCGCAAAGA

>SS--106-3P

TTTGCTGATCGATCCAACCACA

>SS--107-5P

TAAGATATGAGAAATGATGAT

>SS--107-3P

TCATCATTTTTTATATCTTAAG

>SS--108-5P

TGGCCTGGATTTGGTCGCAAATA

>SS--108-3P

TTTGCTGACCATTCCAACCACG

>SS--109-5P

TTTTTCATTACTTTTATAAAA

>SS--109-3P

TTAGCAAAGAGATGAAAAAAGG

>SS--110-5P

TGGTCTTAATGATTGTGGT

>SS--110-3P

TACTACTATCATCAAGACTTCT

>SS--113-5P

TAGGGAAACTGTATTATATATA

>SS--113-3P

TATATAGATACTTTTTTTCTAT

>SS--114-5P

GAATTGGAGGATGATGATA

>SS--114-3P

TCATCATCATTTTCTCCAATCT

>SS--115-5P

GTCTTGGATTGGTCGCAAATT

>SS--115-3P

TTTGCTACCATCACTAGACTG

>SS--116-5P

TCCCCCTTGAAAATCATTAAAT

>SS--116-3P

TTTAATTATTTTCTCGAGGTTA

>SS--117-5P

CTGGCCAGAGACGTTTTGTAGGAT

>SS--117-3P

TCCCAGAACGATTTGGCCAAG

>SS--118-5P

GACCTCTTGGAAACCGGTAAAT

>SS--118-3P

TTTATCCGTTTCCTGAGGTAGA

>SS--119-5P

TTAGAGTCCCATGGTGCCAAAG

>SS--119-3P

ATTGTCAACCTGGGCTCCAGTT

>SS--120-5P

GACGTATCAAAGTGTAGTGGA

>SS--120-3P

TCATTTCACTTTGATATGTTT

>SSCEL-LSY-6-3Z121-5P

ACAAAAATTCACAATCAAAAGT

>SSCEL-LSY-6-3Z121-3P

TTTTGTATTGCTGAATTTTTGT

>SSCEL-MIR-231-3Z122-5P

TAACCTGTCACTCGATGCTTATG

>SSCEL-MIR-231-3Z122-3P

TAAGCTCGTAGCTACAGGTTT

>SSCEL-MIR-790-5Z123-5P

CTGGAGCCCAGGTTGACAATG

>SSCEL-MIR-790-5Z123-3P

TTTGGCACCATGGGACTCTAA

>SS--125-5P

ACCAGGAGCTCCAGGACCACA

>SS--125-3P

TAGACCTGGAGCACCTGGAGC

>SS--126-5P

TGAGATATGGAAAATGATGAT

>SS--126-3P

TCATCATTTTTTATATCTCA

>SS--127-5P

TAAGATATGATAAACGATGACA

>SS--128-5P

TAGTTTGGATACAGTCGCAAAAA

>SS--128-3P

TTTTGCTGATTGATCCAACTGC

>SS--129-5P

AGTGGAATTCTCAAGTGCCAAAAC

>SS--129-3P

TTCGCTTCTTAGAATTCCATTTT

>SS--130-5P

TGAGATATGATAAACGATGACT

>SS--131-5P

ACGGCTGTTATCTGATGCGCCAACC

>SS--131-3P

TGCCTCGATTATCAGCTGACA

>SS--132-5P

TGAGATATGATAAATGATATTTT

>SS--133-5P

ATGGTGGTGGATGAATTACT

>SS--133-3P

TAATTTCTTCATCATTATCT

>SS--134-5P

TTTGAGATATAAAAAATGATG

>SS--135-5P

TAAGCAGTGGTGTGAAGGTTG

>SS--135-3P

ACCTGCGGCACTACTACTTAAA

>SS--136-5P

TGAACTATATCGTGATGATGTA

>SS--136-3P

TACATCGATACCTTCTAGTCCA

>SS--137-5P

GGAGGTGGATGGATTAGTAGTGT

>SS--137-3P

GCCTCATCCTCCTCCTCCTG

P**RECURSOR miRNA sequence**

>STR-MIR-1

CATGCTTCTTTATAGTGCCATATAGCAAGAGAACTTATGGAATGTAAAGAAGTATGCA

>STR-MIR-7880A

CAGTTGGATCGATCAGCAAAAAACGTTTAAAAATTTTTTGCGACTGTATCCAAACTACG

>STR-MIR-50

TGATATGTCTGGTATTCTTGGGTTGATCTGGTCCAGCCGAGTAATATTAGACATATCGAG

>STR-MIR-8365

CACTCCTTTTCGTTTGCTAATATTTGTTTCTAGAAAATATAGGAAATGAATAAGGAGAAT

>STR-MIR-7880B-1

TGGCTTGGATCTTTCTGCAAACATGTTTCAAATACATTTTTGCGACCGAATCCAGGCCACG

>STR-MIR-8366A

ACAGTATTGACTAACGTGTATGTCGTTCTTGGAAGTCCTACCCGTAGTCCAATGCTGTCA

>STR-MIR-7880B-2

TGGTTGGATCGATCTGCAAACATGATATGTAATCGTTTTTGCGACCGAATCCAGGCCACG

>STR-MIR-7880B-3

TGGTTGGATCAGTTTGCAAACATATAATCTTCAAATTTTTTTGCGACCGAATCCAGGCCACG

>STR-MIR-7880B-4

TGGTTGGATCGATCAGCAAATACACATTAGATAATGTCTTTGCGACCGAATCCAGGCCACG

>STR-MIR-7880C

TGGCTGGAGTTGGAGCAAATGTAGTTTTGTATCCTAAATTTGCGACCGAATCCAAGCCTGA

>STR-BANTAM

TGTGATTTCAAATGGACTCCAGATTATTTTATAGACATCTGAGATCATTCTGAAATCACGAT

>STR-MIR-7880D

GATTGGGTCAGGCAGCAAATATACTTTTTTAAGAATGTTTATTTGCGACTGTATCCACATCACT

>STR-MIR-71

TGAAAGACATGGGTAGTGAGACGATGAGTTTGAATTCCGTATCACTATTCTGTTTTTCTCC

>STR-MIR-7880E-1

TGGACTGGATAGTTGCAAACATAATCTTTATTTATTTTTGCGACCAAATCCAGGCCACG

>STR-MIR-7880F

GGCCTGGTTAGTCGCAAAGAATTTTTCAGTGATTCTTTGCGATTGTTTCCAGGCCAGC

>STR-MIR-7880G

GGTCAGATGAGGTCTGCAAATTAATATTCAGAAACTTAATTTGCGACTAAATTTGGACCAA

>STR-MIR-252A

TTAAGTAGTTGTGCCGTAGGTCGTCGTTAATAGTGAATCGACCTACACACGGCTCCTTAAGA

>STR-MIR-7880H

GGTTAGGATCAGTTTGGCAAATATAAAGTTTAAATTATTATTTGCGACTGTATCCAAAACCCA

>STR-MIR-7880I

GGTCTAGTGATGGTAGCAAATTAGTATATTGAAAACTAAATTTGCGACCAATCCAGGACCCG

>STR-MIR-81A

ATGGGTCTATATGATTCTCATGTTAATTGATAATATCATGAGATCATGTTAGACTCATAT

>STR-MIR-8367

GAGTCAAGTAATCTTGGTTAAAAATATCAAATCTCTTTAACCGGATATATTTGACTACAG

>STR-MIR-7880E-2

TGGTTGGAATGGTCAGCAAATATATTTTTATATAAGTATATACATTATTTGCGACCAAATCCAGGCCACG

>STR-LIN-4

TCCCTGAGACTCTACTTGTGACAGATTTAATATGTCCCAAGTTAGATGATCAGGTACC

>STR-MIR-8368

TCCCTGAGATTTCATATAACCGGAGTTTCTGATATATACTCCGTATATGAATGATCAGGTACC

>STR-MIR-236

CGTCATTATCGCAGCTATTAGAGACATTTTGATTGCTTTAATACTGTCAGGTAATGACGCT

>STR-MIR-81B

ATGGGCCTATGTGTGTCTCGTGGTGGATTTTATGATATAGCCATGAGATCATATTAGACCCATCT

>STR-MIR-81C

ATAGGTCTATTTGCTTTCTCATGTTGATTATAATTTCATGAGATCAAATTAGACTTATCG

>STR-MIR-72

AGGCAAGATGTTGGCATAGCTGATAGTCGAAAATTATTCAGTTATGCTATATGTTGCCAGA

>STR-MIR-8369

CTGGAGCCCAGGTTGACAATGCATTACTAAATAGCTTTGGCACCATGGGACTCTAAT

>STR-MIR-7880J

TGGTCGGAATAGTTGCAAATTTTATTCTAATTTTTTAATTTGCGACCTATTTCCAGGCTACT

>STR-MIR-5359

TCTGGGATTGTTTATCTTGGTATCCATGAGATGATACAAGAATAAACATTCCTTGAC

>STR-MIR-86

TAAGTGAATGCTTTGCCACAGTCTTGAATTTAGAATCCTAGCCTGCTGCAACATTCACTTGGC

>STR-MIR-92

AGGTTGGAGCATGCGCCATATTTTTGATAGATTATAATAATATTGCACACGTCCCGGCCTGA

>STR-MIR-7880K

GGTCAGAAATGGTTGCAAATTAGTAGTTTGAAGACTAATTTGTGACCTTTCAATGATCAG

>STR-MIR-8370

AGATGCAGGTTGGGTCTTTATGGAATCTATATCACATAAGGCACAACGTTGGCATCTCT

>STR-MIR-7880L

GGCTTAGGATGGTGCAAATTTATTATTTTTAAATTAATTTGCGACCATATCCAAAGCCCGT

>STR-MIR-7880M

GGTCGGTAGAAGAAGCAAATTAAATATTTTTGTAAATTCAATTTGCGCTCGATACCTGACCAC

>STR-MIR-8371

AGATGGGTGAGTTTGCCAAATTAGTGACTTTAAATACTGAATTTGCGAACGACCCTGTCCAA

>STR-MIR-7880N

TGATGGATAAGGAGCAAATTTGATTGAGAAAACTTTAATTTGCAACTTTATCCATTTCACT

>STR-MIR-252B

TTAAGTAGTAGTGCCGCAGGTTGGAAAATATGATATCACAACCTTCACACCACTGCTTAAT

>STR-MIR-7880O

TGGTTGGATGAGAAGCAAATTTTGTTTTTACTTATTCAATTTGCGACTATATCCAGGCCATT

>STR-MIR-7880P

GGCCAGTGATGTCTCAAGTTCGACTTTGGATCTTAATTTGGGACCATATCTGTGCCAG

>STR-MIR-1175

CGGTGAAGAAGAGAATCTGACTTAGAATATGAATCAAGTGAGATTCTCTTTTCTTCATTC

>STR-MIR-7880Q

GGCTGGTTAGGCTGCAAATCTGGCTTTAGATCATATTTGCGACCAAAATCATGCCAC

>STR-MIR-8372

ACGTTTGATCGAGCTTTAAATAGTATAATTTAAAGCTATTAAAGCTTAGATTAAACGTCT

>STR-MIR-8373

AGAATAACTATATATTATCACCAGTCATAGGAATACTAGTGATATGTACAGTTATTCTGT

>STR-MIR-8366B

ACAGTATTGATATGTGGGTGACAGATTTTAAATTCATGTACCCGTAATCCAATGCTGCAA

>STR-MIR-7880R

TGGCTGGGTTGCTCTGCAAAAATGTTTTTAATATACAGTTTTTGCGACCAATCCAAGCCAAA

>STR-MIR-7880S

TGGCTGGAAAGGCTGCAAATTAAGCTGTTGATTCTAAATTTGCGACCTATATCCAGGCCACG

>STR-MIR-8374

CAACATTCAATTCATATAAAACAATTTTTAAATGTTAAATATGAATGTGAATGTTCT

>STR-MIR-8375

TTTTAAGCATCGGTAACAAACATATTTTTTAAAAATGTTTGGGACCTGTGTTTAAGTAA

>STR-MIR-8376

TACTCAGTACGTAACAAAGATTAATTAAATACAAATATCTTTGCAACGTATCTGGGTTTAC

>STR-MIR-7880T

GCACTTGGTTGGAAGCAAATATGATATAAGTTAATTTTTGTAATTAATTTAAAATATTATTTGCGACCAAATCATGTGCAT

>STR-MIR-9

TCTTTGGTTATTTAGCTATATGAATAATAAGATATTTTTTCATAAGCTGAACGATCAAAGGAA

>STR-MIR-8377

TTTAACGCGGATAGATGAATTGTGATTTGAGCTAAATTCATTGTCCAGCGTTGAAC

>STR-MIR-87A

CATCGGAGACTTTTGGACTCAACCTCAGATTGTAATATGAAGGTGAGCAAGAGCTTCCGGTGTG

>STR-MIR-47

TAGGAGAGCTGTCTTTGTCGGTTGAATATTAGAAAGTCTACTGTCATGGAGTAGCTCTCTTAGA

>STR-MIR-7880T

TGCCCCAGTAAGGCCACAAATTTAGTTTTATAGTTTAATTTGCGACCATTCCAGGGCTA

>STR-MIR-8378

CATTCTGCATTGCTTAATAACTTATCTGATGAATTAGTATTATGCAAAGTAGAATGTA

>STR-MIR-84

TGAGGTAGTGTTAAATATTGTTTTACATGAATTAAACAATATTTTATTCTACTTCATC

>STR-MIR-8379A

AGCTGTCTTTTACAGTATCAGATATATTAAAAATTTTCTAATACTGCCTTAGACGGCTAA

>STR-MIR-76

CGAGTTTCGTGATGTCGAATAAAAGTTTGATAATCTAATTCGTTGTTACTGAAACTCGTC

>STR-MIR-8380

TGGGTATGTTTTTCGTCCAAGATATATTTGATAAAAATCTTGCACGATTTACATACTCACA

>STR-MIR-8381

CAAAAATTCGCAATCAAAAGCTGTCTGATATACATGCTTTTGTATTGCTGAATTTTTGAT

>STR-MIR-8382

TGGAATTCTAAGAAGCGAAACAAGATAATAATAGTTTTGGCACTTAAGAATTCCACT

>STR-MIR-7880V

GGTCGGATGATTATTGCGAATTAGTTATTTTTATACTTAATTTGCGATAACATTCAAGACCAA

>STR-MIR-8383

TCGAATCGTCTAGAAGTTAATTTTGATTGAGATTAAATTGGCTTCGTGATGATTCAAA

>STR-MIR-7880W

GACAGGAATTCTAATTGCAAATTGATTTTTTTAAATGTCAATTTGCAAAGTATATCCAAGTCGA

>STR-MIR-8384

ATGGATCTTTGTATGTCTCATGTACAAGAGATTCATGAGATTATGAAGATTCATTA

>STR-MIR-8385

TTTGCAAAGTATATCCAAGTCGATAGTGTTTCATTATCAATCAACAAATTTTAATATTAAAATACTCGTGGCTGGAAAGGCTGCAAATT

>STR-MIR-8386

CGGGTTTGTTACTTGTTGAATTTAATAAAAATAAAAATTCACCGGGTAGACAAACGCGCG

>STR-MIR-8387

AAATTTCATGATTCGGTAGGAACTGAATAGAAAATGTTTCACCGAAGTCATGAAGTTTC

>STR-MIR-40

AGAGTTTGTCGGCATGGTGACTTATAATTGTTTAAGTAATTCACCGGGTGTACGAATTCTCA

>STR-MIR-124

CGCTTTCATCCGTGACTTTAGAAGAGTCTTATATCACTAAGGCACGCGGTGAATGC

>STR-MIR-184

ACTTATCAACTTCATGCCTTGTTTAAAATTTTTATAAAACTGGACGGAAGTTTGATAAGAGA

>STR-MIR-8388

AAGCAAAATGGGATGCAAATAAATATAATAATAAAATTTGAGACCTCATCTTAGCTTAG

>STR-MIR-8389

AATATCTGAATTGTTTACAAAACTATATGATTAAAAAGTCATGTAAACATTTTAGATATACA

>STR-MIR-240

AGAAGTTTGGTAGTCATGAACATAATACTTTTAAAATGTACTGGCCTTCAAACTTCTTA

>STR-MIR-8390

TATTTCAAAAGTTGCATATGACAAGATTTGTTCCATTGTAAATGCATCTTTTGAGATAGA

>STR-MIR-8391

TTGTGGAGTCGTGGAAAAATGTAGAATATGAACTCATCAATCCTCGTACTTCACAACA

>STR-MIR-8392

AACTCTTGATTTCTGTAGAATTTTGTTACATTTCTTAATTCACCGGAAACCAGGAGAGG

>STR-MIR-37A

AGTGAATGTTACTTCGGTAATGGAATTTTTGAAATCACATCACCGGGTATACATTCATCAA

>STR-MIR-255

GTAAGAAGTCAAATCGGTTTCAGAGATGTTCTTAAACTGAATTGATTCCTTACAG

>STR-MIR-8393

AGCATTTGAATCATCGATGATTAATCAAATGAGTTACTCACCGGGATATCAGATGCTC

>STR-MIR-360

TTGTGACCGTTGTAACGGCTATTCAATATTAGTATAAAAGAATGTCGGATACAATGAGCACAAA

>STR-MIR-8394

AAGGTGTGGTCGTCGCCATTATATCGACTGAAGTGATATATGGCACGTACACCATCTTC

>STR-MIR-58

ATGACACTGAATGAACTGACGTATCAAGAGAATCGTGAGATCATATTAGTGTCATTT

>STR-MIR-37B

AAGTAATTGTTTCCATGTTGATTTATTGTTAAATAACTCACCGGGAATACAATTACCAAT

>STR-MIR-8379B

TAGCCGTCTAAGGCAGTATTAGAAAATTTTTAATATATCTGATACTGTAAAAGACAGCTAA

>STR-MIR-279

CAAGGTAATTTCATAGTCATGGTTTATTGACAGGCGCATGACTAGAACATTACTTTG

>STR-MIR-87B

CGTTAAACATTGGCTCAACATCTAATTGATATAATGTTGTTGAGCAATGTTTTAATGCA

>STR-MIR-2

TTCAAAAGACTGTGTTTCAGGATATGTAAATTATGTATCACAGTTATTTTGAATCTG

>STR-MIR-234

ATAGGTATTCTCAAACAATTGCATTATCTTATAAATTGTTATTGCTTGAGAATGCACAT

>STR-MIR-7880X

TGGCCTGGATTCGGTCGCAAAGACATTATCTAATGTGTATTTGCTGATCGATCCAACCATG

>STR-MIR-8395

ACGACCTTATTTTGTCTTCAACATAATTTTAGAGATGTTTAAGGCATTTTAAGGTTGTTC

>STR-MIR-8396

CGGGTTTATCATTGCCAAACTCAAAAGCGTTTTGTAAGTTTGGCACTGTATAGGCTCCAA

>STR-MIR-34A

TGGCAGTGTGGTTAGCTGGTTGTGATTTTTTAGACGCTCAACAGCTCACTCAACTGCCAAG

>STR-MIR-34B

TGGCAGTGTAGTTAGCTGGTTGATGTTTTAAAAATCAACAGCTTACCAAATTGCCATC

>STR-MIR-8397

AGGTGTAGATTGCCATTCAACTGTTAAGAAAAAACTGTTGAAAGACGTTCTATATCTCT

>STR-MIR-8398

AGTTCTTTCTTTTTAGCTTTAGTATTATAATGTATATTAAGCTGCCATGAAAGAACTAA

>STR-MIR-8399

TTTCAGTAGGGACTTTATTAAAACTAAAAGAAAGTAATTTAATGATGTCTTACGGAAATA

>STR-MIR-8400

TGTGGGTTAAATCATCTAGATTCATAGATTAACATACGATTCTAGGTAATTTAAGCTACAAA

>STR-MIR-8401

TTGAATCATCACGAAGCCAATTTAATCTCAATCAAAATTAACTTCTAGACGATTCGAAT

>STR-MIR-7880Y

GGTCAGATGATGTCCACAAATTAGTATTTAAGAAACTTAATTTGCGACTAAATCTAGACC

>STR-MIR-8402

TCAAAAATTCAGCAATACAAAAGCATGTATATCAGACAGCTTTTGATTGCGAATTTTTGTAA

>STR-MIR-34C

TGGCAGTGTGATTAGCTGGTTGAAGAAGATTATAAATTCAACAGCTCATTAAACTGCCTCT

>SS--28

AGATGGGTGAGTTTGTCAAATTAGTATTTTTAGAGGCTAAATTTGCGAACGATCCTGTCCAA

>SS--29

TGGTCGGGTGAGAAGCAAATTTTGTTAATATTTCTATAATTTGCGACTATATCCAGACCATT

>SS--30

GGTTAGGATCAGTTTGGCAAATATAAAATTTAAAATATTATTTGCGACTGTATCCAAAACCCG

>SS--31

TGGTTGGATCGATCAGCAAATACATATTAGATAATGTCTTTGCGACCGAATCCAGGCCAC

>SS--32

AATTGGTTAGGCTGCAAATCTGGCTTTAGATCAAATTTGCGACCAAAACCAAGACA

>SS--33

AGTCTAGTGATGGTAGCAAATTAGTATGTCAAAAATTAAATTTGCGACCAATCCAAGACTGA

>SS--34

GGTCAGATGATGTCTGCAAATTAGTATTTTTAAAAACTTAATTTGCGACTAAATCCAGACCAA

>SS--35

TGGCTGGAGTTGGAGCAAATATAGTTTTGTATCATAAATTTGCGACCAAATCCAAGCCTG

>SSCEL-MIR-80-3Z36

GTGGTTTCAAATGAACTCCAGATTATTTTATAGACATCTGAGATCATTATGAAATCACG

>SS--37

GACAGGGAGTATATTTGCAAATTCATATTAAAATAGTCAATTTGCAAAGTATACCCAAGTCGA

>SS--39

ATGGATCTTTGTATGTCTCATGTACAAGAGATTCATGAGATTATAAAGATTCATTA

>SS--40

GGTCAGATAAAGTCTGCAAATTATTATTTAAAAAGTTAATTTGCGACTAAATCCCGACCAA

>SS--41

AGCCGGATCTTTAGCAAATTAACAAATTGAAATGTAATTTGCAGATAGATTCCAGCCTG

>SSCEL-MIR-80-3Z42

ATGAGCCTATTTGTGTCTCGTGTGCATTAAGAACTCATGAGATCAAACTAGACTCATT

>SSCEL-MIR-75-3Z43

CTTTGGTGATTTAGCCGTAATGAGCATTTTTGGAACTCATAAAGCTAGGTTACCAAAGCT

>SSCEL-MIR-60-3Z44

CATTCTACATTGCTTAATAACTTATCTGTTAAATTAGTATTATGCAAAGTAGAATGC

>SSCEL-MIR-80-3Z45

ATGGCACTGGATGAGCTGACGTATATAAAAGAATCGTGAGATCACATCAGTGTCATTT

>SSCEL-MIR-232-3Z46

TGTGTCAGTACTTGCATTTACATTTTTTTTATATATTTGTAAATGCATCTCACTAGCACAA

>SSCEL-MIR-124-3Z47

AGATGCAGGTTGGTTCTTTATGGAATCTTTATCACATAAGGCACAACATTGGCATCTCT

>SS--48

TACTCAGTACGTAACAAAGATTAATTTAAATCGAATTTCTTTGCAACGTATCTGAGTTTAC

>SSCEL-MIR-228-5Z49

CACGCGTCAATCGTAGCCATCTCTGATCTGAACCATGAAATGGCACCTTTGAACGTGTGC

>SS--50

TGGCCGGTAAGGGCGCAAATAAAGCATTAAGTTCTTAATTTACGACCTAATCCGTGTCATT

>SS--51

ACTTATCAACTTCATGCCTTGTTTAAAATTTAATAAAACTGGACGGAAGTTTGATAAGTGA

>SSCEL-MIR-35-3Z52

AGCATTTGAATCATCGATGATTAATCTAGTGAATTATTCACCGGGATATCAAATGCTA

>SSCEL-MIR-360-5Z53

TTGTGACCGTTGTAACGGCTGTTCAATATTTAAAAAAAAAGAATGTCGGATACAATGAGCACAAAA

>SS--54

TGGCCAGTGATGTCTCAAGTTTGACTTTAGATTTTAATTTGGGACCATAATTGTGCCAGT

>SSCEL-MIR-60-3Z55

CTTTGTAGCAAGCACAATTACTTATCTTTAAAAAAAAAGTATTATGCCTGACTACAAAGTCT

>SS--56

GATTCGATGATTACGCAAATTTAGTCAATTGAAACATTAGAATTTGCGTATAAATCCTTGAATCTC

>SS--57

AACCTTAAGAAGACGGCTAACTTTAATTTTGAATAAAGTTGTCTACTTCAAGAGGAAGA

>SS--58

AACCTTAAGAAGACGGTCAACTTTATATTTGAATAAAGTTGTCTACTTCAAGAGGAAGA

>SS--59

GGTCGGTAGAAGAAGCAAATTAAATAATTATATAAATTCAATTTGCGCTTGGTACCTGACTAC

>SSCEL-MIR-35-3Z60

AGTAATTGTTTCCATGTTGAGTTGTTTAGTAGTAACTCACCGGGAATGCAATTACTAG

>SSCEL-MIR-790-5Z61

TGGAATTCTAAGAAGCGAAACAAAAATATGACAGTTTTGGCACTTGAGAATTCCACT

>SS--62

TACCTCAGGAAACGGATAAATTTAGTTTTTAAGTTAATTTACCGGTTTCCAAGAGGTCG

>SSCEL-MIR-790-5Z63

AGGGTTTAACTTTGCCAAACTTGACTATTCTAATAGTTTGGCACAGTATAGGCTCCAA

>SS--64

TGAGATATAGAAAATGATGACAAAGTATTGAGCATGAAAGAATCATCGTTTATCATATCTCAAG

>SSCEL-MIR-234-3Z65

AGGTATTCTCAAACAATTGCATTATCTTATAAAATGTTATTGCTTGAGAATACATAA

>SS--66

TTGTGGAGCTGTGGAAAAAGTGTGAATTTTGAACTCATAAATCCTCGGCTTCACAAT

>SSCEL-MIR-87-3Z67

ACGTTAAACATTGGCTCAACATCGAATTGATATAAAGTTGTTGAGCAATGTCTTAATGCAA

>SS--68

AGGTTGGATATTGTTATCAAATTAGTATTTTAAGAACTGAATTTGGGAACAAATCCAATACCTT

>SS--69

TGAGATATGGGAAATGATGACATATGGTTGAGCTTGAAAAAGTCATCGTTTATCATATCTTAAG

>SS--70

AACCTCGAGAAAATAATTAAACTTAATTTTAAATAAATTTAATGATTTTCAAGGGGGAG

>SS--71

TGAGATATAGAAAATGATGACATATGACTGAGCTTGAAAAAGTCATCGTTTATCATATCTTAAG

>SS--72

TGAGATATAAAAAATGATGACAAGTGATTAAGCATGTAGATGTCATCGTTTATCATATCTTAAG

>SS--73

TGAGATATAAAAAATGATGACATATGATTGAGCTTGAAAAAGTCATCGTTTATCATATCTTAAG

>SS--74

TGAGATATAGAAAATGATGACGTATGGTTGAGCTTGAAAAAGTCATCGTTTATCATATCTTAAG

>SS--75

TGAGATATGAAGAATGATGACAAATGATTAAGCACGCAAAATCATCGTTTTTCATATCTTAAG

>SSCEL-MIR-2-3Z76

TGATTCAAGGGACTGTGTTTCAAGAAATGTTAAAGATGTATCACAGTTTTCTTGAATTTG

>SS--77

TGTTGGATAGATCAAAAAAAACCCCTCGCTGCACGTTTTATTTTGATCTACCCAACAT

>SS--78

TGATTTTTTTTTATTTGATAAAGTTAGGCAATCCTCTATCAGATAAAAAAAAATCATAA

>SS--79

AACCTTAAGAAGACGGCCAACTTTAATTTTGAATAAAGTTGTCTACTTCAAGGGGAAG

>SSCEL-MIR-71-5Z81

AGATATTATTTGTCGGTCAACAGGTTATATAAGATAAAAGCCGTTGAAAGACAAATTATATCTTT

>SS--82

TGAGATATAAAAAATGATGACAATTAGTTTATCAAAGAAAAATCATCGTTTCTTATATCTCAAG

>SS--83

TGAGATATAGAAAATGATGACGTGTTATTGATGCGCACAATTTTCATCGTTATCATATCTCAAG

>SS--85

AACCTTAAGAAGACGGCCAACTTTAATTTTGAATAAAGTTGTCTACTTCAAAAGGAAGA

>SS--86

TGAGATATAGAAAATGATGACATATGGTTGAGCTTGAAAAAGTCATCGTTTGTCATATCTTAAGA

>SS--87

TGAGATATAGAAAATGATGACAAACGATTGAGTATAAAGAAATCATCATTTATCATATCTCA

>SS--88

CTTAAGATATAAAAAATGATGACAATTAGTTTATTAAAGAAAATCATCATTTCTCATATCTTAAGA

>SS--89

TAAGATATAGAAAATGATGATAATTAGTTTATCAAAGAAAAATCATCATTTCTTATATCTTAA

>SS--90

TTGACCAGGTTTGAAAGAAAAAATACATACATTTTTTTCTGTCAACCCCGGTCGAA

>SS--92

TCAATTTTGCTACTCTTCAAATTATTTATAAATTCATTTGAAGTATAGTAAAAGTTTGAT

>SSCEL-MIR-1820-5Z93

AAAAATTCAGCAATACAAAAGTATGTATATCAAACAACTTTTGATTGTGAATTTTTGT

>SS--94

AACCTTAAGAAGACGGCCAACTTTAATTTTTAATGAAGCTGTCTACTTCAAGAGGAAGA

>SS--95

GTCGTTCAAAAATAAATGTCGCAAAAAAGATTAGTGAGGGATATAAAAATACGACATTCATTTTTGAACGACCT

>SS--96

CGTGATTTCATAATGATCTCAGATGTCTATAAAATAATCTGGAGTTCATTTGAAACCACAAT

>SS--97

TAAGTAATTATAGTAAGGAAATTATTAAAAATTTTCCTTAATATAAAATTACT

>SS--98

TTTGTAGTCAGGCATAATACTTTTTTTTTAAAGATAAGTAATTGTGCTTGCTACAAAGTCT

>SS--99

AACCTTAAGAAGACGGCCAACTTTAATTTTGAATAAAGTTGTTTACTTCAAGAGGAAG

>SS--100

AAACCTGTAGCTACGAGCTTATAGTTTTAAAAATATCTTCATAAGCATCGAGTGACAGGTTAA

>SS--102

TTAACCGGGTTTAAAAGAAAAAATACATACATTTTTTTCTGTCAACCTCGGTCGAAA

>SS--103

TGGCCTGGATTCGGTCGCAAAAATGATTTTTTTACCATGTTTGCAGATCGATCCAACCACG

>SS--104

TAAATTGAAGATGGAGTATCTTGAAAGTTTAAGATACTTCACTTGTCAATTTAATT

>SS--105

GGTCTGGATTTAGTCGCAAATTAAGTTTTTAAAAATACTAATTTGCAGACATCATCTGACCAA

>SS--106

TGGCCTGGATTCGGTCGCAAAGACATTATCTAATATGTATTTGCTGATCGATCCAACCACA

>SS--107

TAAGATATGAGAAATGATGATTTTCTTTAATAAACTAATTGTCATCATTTTTTATATCTTAAG

>SS--108

TGGCCTGGATTTGGTCGCAAATAATGTATGTATATTTATAATGAATATATTTGCTGACCATTCCAACCACG

>SS--109

TTTTTCATTACTTTTATAAAAAAAAATTATTCTTTTTTTTAGCAAAGAGATGAAAAAAGG

>SS--110

TGGTCTTAATGATTGTGGTGTTGTTGTTACTGAAATACTACTATCATCAAGACTTCT

>SS--113

TAGGGAAACTGTATTATATATATGATCATTTATACACATATATATAGATACTTTTTTTCTAT

>SS--114

GAATTGGAGGATGATGATAGGGGTGTCGAAGATTTGATAGAAGATTCAATATCATTAAAATTACCCTCATCATCATTTTCTCCAATCT

>SS--115

GTCTTGGATTGGTCGCAAATTTAATTTTTGACATACTAATTTGCTACCATCACTAGACTG

>SS--116

TCCCCCTTGAAAATCATTAAATTTATTTAAAATTAAGTTTAATTATTTTCTCGAGGTTA

>SS--117

CTGGCCAGAGACGTTTTGTAGGATTAATCTTAACTACTCCCAGAACGATTTGGCCAAG

>SS--118

GACCTCTTGGAAACCGGTAAATTAACTTAAAAACTAAATTTATCCGTTTCCTGAGGTAGA

>SS--119

TTAGAGTCCCATGGTGCCAAAGCTATTTAACACAACGCATTGTCAACCTGGGCTCCAGTT

>SS--120

GACGTATCAAAGTGTAGTGGATAATATACGATATCATTTCACTTTGATATGTTT

>SSCEL-LSY-6-3Z121

ACAAAAATTCACAATCAAAAGTTGTTTGATATACATACTTTTGTATTGCTGAATTTTTGT

>SSCEL-MIR-231-3Z122

TAACCTGTCACTCGATGCTTATGAAGATATTTTTAAAACTATAAGCTCGTAGCTACAGGTTT

>SSCEL-MIR-790-5Z123

CTGGAGCCCAGGTTGACAATGCGTTGTGTTAAATAGCTTTGGCACCATGGGACTCTAA

>SS--125

ACCAGGAGCTCCAGGACCACAAGGAGCACCTGGAAAACCTGGACGTCCTGGTAGACCTGGAGCACCTGGAGC

>SS--126

TGAGATATGGAAAATGATGATTTTGCGCGCTTAATAACTTGTCATCATTTTTTATATCTCA

>SS--127

TAAGATATGATAAACGATGACATCTACATGCTTAATCACTTGTCATCATTTTTTATATCTCA

>SS--128

TAGTTTGGATACAGTCGCAAAAAATGTTTGAACATTTTTTTGCTGATTGATCCAACTGC

>SS--129

AGTGGAATTCTCAAGTGCCAAAACTGTCATATTTTTGTTTCGCTTCTTAGAATTCCATTTT

>SS--130

TGAGATATGATAAACGATGACTTTTTCACGCTCAATTATTGGTCATCATTTTTTATATCTCA

>SS--131

ACGGCTGTTATCTGATGCGCCAACCCAACCATTATGTCGATTGGTGCCTCGATTATCAGCTGACA

>SS--132

TGAGATATGATAAATGATATTTTTTTACGCTTCAGCCATTTGTCATCATTTTTTATATCTCA

>SS--133

ATGGTGGTGGATGAATTACTGTATTTTCTACCAAAGTAATTTCTTCATCATTATCT

>SS--134

TTTGAGATATAAAAAATGATGACCAATAATTGAGCGTGAAAAAGTCATCGTTTATCATATCTCAAG

>SS--135

TAAGCAGTGGTGTGAAGGTTGTGATATCATATTTTCCAACCTGCGGCACTACTACTTAAA

>SS--136

TGAACTATATCGTGATGATGTATCAAGTCCAAGTTTTAAGGAAAGTGATACATCGATACCTTCTAGTCCA

>SS--137

GGAGGTGGATGGATTAGTAGTGTTACTAAATAATGAATTTACCAACGCCTCATCCTCCTCCTCCTG
